# Supplementary material for: Monitoring and modeling of lymphocytic leukemia cell bioenergetics reveals decreased ATP synthesis during cell division
Source: Nat Commun. 2020 Oct 5;11:4983. doi: 10.1038/s41467-020-18769-y (PMC7536222; doi:10.1038/s41467-020-18769-y)
Supplement: Supplementary file 1 — Supplementary information [file 41467_2020_18769_MOESM1_ESM.pdf]

Supplementary Information for

Monitoring and modeling of lymphocytic leukemia cell bioenergetics reveals decreased ATP synthesis during cell division

Joon Ho Kang, Georgios Katsikis, Zhaoqi Li, Kiera M. Sapp, Max A. Stockslager, Daniel Lim, Matthew G. Vander Heiden, Michael B. Yaffe, Scott R. Manalis, Teemu P. Miettinen

Correspondence to: teemu@mit.edu

This PDF file contains:

Table of Contents
Supplementary Notes 1-9
Supplementary Figures 1-15
Supplementary References

Table of Contents

Supplementary Note 1. Non-invasive, continuous, high-resolution monitoring of mass normalized fluorescence signals in single-cells 3

Supplementary Note 2. TMRE as a probe for ΔΨm 4

Non-quenching and quenching mode experiments with ΔΨm responsive probes 4

Influence of cell mass, volume and mitochondrial content on TMRE signal in mitosis 4

Influence of ΔΨp on TMRE signal in mitosis 5

Characterizing TMRE diffusion dynamics and TMRE detection accuracy 5

Supplementary Note 3. Modeling mitochondria as an electrical circuit 7

Supplementary Note 4. Derivation of analytical solution to electric circuit model 10

Supplementary Note 5. Obtaining time constants (RCs) by fitting analytical solutions to experimental data 12

Converting TMRE to voltage 12

Defining fitting regions 12

Data exclusion from the model 13

Supplementary Note 6. Calculating ATP current (I_{ATP}) from time constants (RCs) 14

Approach for calculating $IATP$ currents 14

$IATP$ for $CDK1_{off}$ 14

$IATP$ for $CDK1_{on}$ 16

General implications of RC values 17

Supplementary Note 7. Calculating ratios and integral of current $IATP/C$ 19

Supplementary Note 8. Error analysis 20

Case $CDK1_{off}$ 20

Case $CDK1_{on}$ 21

Calculating errors for currents I_{ATP}/C, its ratios and integrals 23

Supplementary Note 9. Sensitivity analysis for model 24

Supplementary Figures 26

Supplementary References 49

Supplementary Note 1. Non-invasive, continuous, high-resolution monitoring of mass normalized fluorescence signals in single-cells

We wanted to study the cell cycle dependent behavior of mitochondrial bioenergetics. To do this, we sought for a method that i) allows continuous monitoring of single-cell fluorescence reporters, such as mitochondrial membrane potential (ΔΨm) sensitive fluorescent probes, ii) enables fluorescence measurements with high temporal resolution and precision, iii) is non-invasive by minimizing phototoxicity, iv) allows cell size normalization of the fluorescence signals. To achieve this, we utilized a microfluidic mass sensor called the suspended microchannel resonator (SMR). SMR quantifies the buoyant mass of a single-cell non-invasively as the cell flows through a vibrating cantilever[1-4]. Using a hydrodynamic trapping approach, the cell can be repeatedly measured over multiple cell cycles, where one of the daughter cells is randomly discarded and the other is monitored following each division. We coupled the SMR to a fluorescence detection setup, where the excitation laser would only be turned on for a brief period during which the cell passes through the laser and the total fluorescence emission is quantified using photomultiplier tubes (Supplementary Fig. 1a, b, Methods). This approach allowed us to monitor mass normalized fluorescence signals of single-cells with a typical temporal resolution of 2 min over multiple cell cycles without perturbing normal cell growth (Fig. 1a)[2,3,5]. Detailed characterization of our mass measurement precision (measurement error < 0.1 pg for a typical L1210 cell) is previously published[3] and details of our fluorescence measurement precision can be found below (Supplementary Note 2).

Supplementary Note 2. TMRE as a probe for ΔΨm

We selected TMRE as our main proxy for ΔΨm due to TMRE's low mitochondrial binding, low electron transport chain inhibition and fast equilibrium[6-10]. As lipophilic cation, TMRE accumulates in mitochondria based on the negative charge in mitochondrial matrix. However, the cytosol also has a slightly negative charge compared to the extracellular media. Thus, while TMRE accumulates primarily to mitochondria and the total TMRE signal reflects primarily ΔΨm, some TMRE can also accumulate in the cytosol and bias the total TMRE signal intensity as a probe for ΔΨm[7-9,11,12]. Below, we show in L1210 cells that i) mitotic increase in TMRE signal reflects an increased ΔΨm, ii) mitotic changes in plasma membrane potential (ΔΨp) or cell volume do not significantly contribute to the TMRE signal, and iii) TMRE diffusion speed or our fluorescence detection accuracy do not bias our quantifications of TMRE dynamics.

Non-quenching and quenching mode experiments with ΔΨm responsive probes

In all our experiments with TMRE, we used TMRE in non-quenching concentrations (10 nM). In a non-quenching mode, increased ΔΨm results in increased TMRE accumulation in mitochondria and, consequently, increased TMRE fluorescence from the cells. We validated this TMRE behavior by increasing ΔΨm using oligomycin (an inhibitor of ATP synthase) and by reducing ΔΨm using FCCP (a protonophore) (Supplementary Fig. 2a).

ΔΨm responsive probes, such as TMRE or Rhod123, can also be used in very high concentrations where the probes switch to quenching mode [6]. In the quenching mode, the ΔΨm probe accumulates to mitochondria in such a high concentration that the fluorescence signal is quenched. Thus, increased ΔΨm results in increased probe accumulation in mitochondria which further quenches the probe fluorescence, resulting in decreased total fluorescence from the cells. We validated this behavior for 10 μM Rho123 using oligomycin and FCCP (Supplementary Fig. 2b). Notably, in the quenching mode experiments, the cell culture media does not have Rhod123. Thus, any changes in the total cell fluorescence, as seen in mitosis (Fig. 1b, Supplementary Fig. 4a), cannot be explained by artificial uptake of the probe or by changes in plasma membrane potential. Together with the TMRE measurements in non-quenching mode (Fig. 1a), these results indicate increased ΔΨm in mitosis.

Influence of cell mass, volume and mitochondrial content on TMRE signal in mitosis

Changes in cell size and mitochondrial content can affect the TMRE accumulation in the cell and, consequently, the TMRE signal. We normalized our TMRE measurements to the buoyant mass of a cell, enabling us to account for changes in cell mass observed throughout the cell cycle (Fig. 1a). Importantly, in early mitosis, when TMRE signal increases, cells undergo mitotic cell swelling, which increases cell volume, but has little influence on buoyant mass[1,3,13]. To exclude the possibility that this volume increase is responsible for the TMRE increase, we inhibited the mitotic cell swelling, and consequently the volume increase, using EIPA[1]. By doing so, we observed that the mitotic TMRE increase was not dependent on the volume increase

(Supplementary Fig. 4c-e). This may reflect the relatively small cytosolic volume present in L1210 lymphocytes, which reduces the influence that cytosolic volume has on TMRE accumulation. Furthermore, FL5.12 cells, which also increase their volume in mitosis[1], did not display TMRE increase in mitosis (Supplementary Fig. 3c). We also examined how mitochondrial content changes during mitosis by monitoring of the ΔΨm-insensitive MitoTracker Green probe. As mitochondrial content is known to scale with cell size[14-16], we normalized the MitoTracker Green signal to cell's buoyant mass. The mass-normalized MitoTracker Green signal did not change during the mitotic mitochondrial hyperpolarization (Fig. 1c, Supplementary Fig. 4b), indicating that mitochondrial volume does not radically alter during mitosis. Thus, changes in cytosolic or mitochondrial volumes don't explain the TMRE behavior in mitosis.

Influence of ΔΨp on TMRE signal in mitosis

Non-quenching mode experiments with ΔΨm responsive probes can be affected by changes in ΔΨp. To assess how ΔΨp changes in mitosis, we monitored the ΔΨp responsive probe DiBAC$_4$(3) and normalized its fluorescence signal to the cell's buoyant mass. We observed a decrease in DiBAC$_4$(3) signal during early mitosis (Supplementary Fig. 4c), suggesting an increase in ΔΨp. However, the ΔΨp increase occurred earlier than TMRE increase, similarly to the mitotic cell swelling (Fig. 1e), suggesting that the ΔΨp change has only limited influence on TMRE signal. We then inhibited mitotic cell swelling using EIPA and observed that the EIPA treatment reduced the DiBAC$_4$(3) change (Supplementary Fig. 4c, d). Importantly, inhibition of mitotic swelling, and thus of ΔΨp increase, did not reduce the TMRE increase in mitosis (Supplementary Fig. 4e). Thus, the extent to which plasma membrane hyperpolarizes is not enough to significantly affect TMRE signal. This is consistent with our other observations that i) quenching mode experiments with Rhod123 display increased ΔΨm (Fig. 1b, Supplementary Fig. 4a), and ii) the inhibition of mitochondrial ATP synthase with oligomycin significantly influences the mitotic TMRE dynamics (Fig. 4d). In conclusion, while changes in ΔΨp will always influence TMRE signal, the mitotic TMRE increase in L1210 cells reflects primarily changes in ΔΨm.

Characterizing TMRE diffusion dynamics and TMRE detection accuracy

We also considered the limits of our approach in detecting the precise kinetics of mitotic TMRE increase and decrease. Our observations of TMRE dynamics are limited by our measurement precision and frequency, and the diffusion rate of TMRE in response to changes in ΔΨm. We first quantified our TMRE measurement precision. To account for both the technical error in our measurements and the biological variability in mitochondrial localization, which can also influence the total fluorescence detected, we labelled mitochondria using MitoTracker Red CMXRos, fixed the cells, and repeatedly measured individual cells to quantify the error for each fixed cell. This indicated that our measurement error is dependent on the total fluorescence intensity (Supplementary Fig. 1c) and for a typical mitotic L1210 cell our TMRE measurement error is approximately 2% (Supplementary Fig. 1d). The typical frequency of our TMRE measurements when monitoring live cells in mitosis was 2 min. Thus, our temporal resolution was

approximately 4 min according to the Nyquist rate. In conclusion, TMRE signal change during the mitotic mitochondrial hyperpolarization (~50% increase in TMRE signal over ~30 min period) is an order of magnitude larger that our TMRE measurement precision and temporal resolution.

Next, we assessed if TMRE diffusion speed is limiting our ability to detect the full dynamics of mitotic mitochondrial hyperpolarization. TMRE is known to diffuse across cell membranes rapidly, reaching equilibrium in minute timescales, especially in small cells with high surface-to-volume ratio[7,8,17,18]. We took two separate experimental approaches to validate that TMRE diffusion rate is fast enough to capture the ΔΨm dynamics during mitosis. First, we monitored mitotic TMRE behavior in the presence of 1 μM tetraphenylborate (TPB). TPB is known to facilitate the diffusion of tetramethylrhodamidine esters through the plasma membrane, thus increasing the rate at which equilibrium is reached[7,8]. We did not observe changes in mitotic TMRE dynamics in the presence of TPB (Fig. 2c). Second, we monitored TMRE signal decay rate on a population level following mitochondrial uncoupling using 5 μM FCCP (Supplementary Fig. 2d). We used this data to quantify exponential time constants for TMRE signal loss (i.e. TMRE diffusion speed out of the cells). We then compared the time constants obtained after FCCP treatment to time constants obtained by fitting an exponential decay to the TMRE depolarization observed in late mitosis (Fig. 4d). Depending on the data fitting used to derive the time constant values, TMRE diffusion speed was 1 or 2 orders of magnitude faster than the TMRE signal change in mitosis (Supplementary Fig. 2e). Thus, in the mitosis of L1210 cells, TMRE signal is a reliable proxy for capturing the ΔΨm dynamics.

Supplementary Note 3. Using an electrical circuit model to quantify mitochondrial bioenergetics

Our experimental evidence shows that the mitochondrial hyperpolarization in mitosis takes place during prophase, prometaphase and metaphase (Fig. 1), which correlates with the timing of CDK1 activity[19]. Consistently, we show that CDK1 activity is required for the mitochondrial hyperpolarization (Fig. 2). Importantly, Cyclin B-CDK1 activity is regulated in a switch-like manner that causes the system to operate in fully active or inactive state with brief transition periods between these two stages[19-21]. Consistently, our data show that arresting cells in metaphase, where CDK1 is active, results in a new, stable level of mitochondrial polarization (Fig. 2a). Together, these results indicate that the cells switch their metabolic state when passing through the G2/M transition (CDK1 turns on) and revert back to the original state when passing through the metaphase/anaphase transition (CDK1 turns off).

We reasoned that this switch-like (biphasic) behavior of CDK1 activity driving mitochondrial hyperpolarization would allow us to develop a quantitative model of mitochondrial bioenergetics in mitosis. To achieve this, we utilized an electrical circuit model of mitochondria (Fig. 4a) which is consistent with the common 'electrical circuit' view of mitochondria[9,18,22-27]. Our electrical circuit model takes as input the experimental time-series data of TMRE signal of individual cells and converts this to ΔΨm (Supplementary Note 5). By processing the resulting time-series of ΔΨm, the model outputs the electrical current corresponding to ATP synthesis. Thus, in our model ΔΨm is the sole driver of the proton motive force (Δp) used for ATP synthesis. Note that in mammalian cell mitochondria ΔΨm is the dominant component of Δp and ΔΨm is also considered indispensable for ATP synthesis[9,12,18,23,28,29].

Our model is formed of components describing the electrical circuit. The voltage (V) across the circuit represents ΔΨm. The voltage source (E_{ETC}) with internal resistance (R_{ETC}), which together form a single 'battery', represent the electron transport chain (ETC). We build our model so that the E_{ETC} has a voltage that is higher than what can be generated across the inner mitochondrial membrane. Consequently, this 'battery' that represents the ETC generates a current I_{ETC} that flows only in one direction, representing the pumping of protons out of mitochondrial matrix (Fig. 4a) (please see discussion below). Thus, the current I_{ETC} representing the ETC activity is regulated solely by the R_{ETC}. The formulation of our model to only have one-directional proton movement through the ETC is equivalent to assuming that net proton flow through ETC (i.e. the sum of all ETC activity in all mitochondria in a cell) is in forward direction during mitosis. Although reverse flow through ETC is physiologically possible[30,31], in order for reverse ETC to take place redox metabolite ratios have to change significantly[30]. More importantly, our experiments show that even in the presence of oligomycin, mitochondria can further hyperpolarize in mitosis, indicating that the net proton flow through ETC is not reversed in early mitosis. However, this does not exclude the possibility that reverse net flow through ETC takes place briefly following metaphase-anaphase transition, slightly influencing our results. Note also that in

our model I_{ETC} represents the transport of any charged ions or molecules that increase the voltage across the inner mitochondrial membrane.

The ATP synthase is represented in our model as a resistance R_{ATP} and the leakage across the membrane is represented as R_{LEAK}. The currents through these components are I_{ATP} and I_{LEAK}, respectively. Note that I_{LEAK} also represents the transport of other charged ions or molecules that decrease the voltage across the inner mitochondrial membrane without producing ATP. Our model also accounts for the well-known non-ohmic ΔΨm-dependent leakage[23,32,33] when deriving ATP synthesis rate, I_{ATP}.

Our electrical circuit model takes into account the fact that mitochondria do not hyperpolarize immediately but within some time scale from the activation of the switch-like activity of CDK1. We do this by including a capacitance (C), as the inner mitochondrial membrane is an effective insulator, largely impermeable to ions, acting as a charge storage. We set the capacitance of our circuit to be constant throughout mitosis. To our knowledge, measurements of mitochondrial inner membrane capacitance do not exist, but it is known that a capacitance can change if the membrane area and/or composition (i.e. membrane thickness and permeability) change. It is highly unlikely that the change in membrane capacitance fully drives the change in ΔΨm, as the dynamics that we model are rapid (~20 min increase and ~20 min decrease in ΔΨm) (Fig. 1). For changes in capacitance to explain these dynamics, there would have to be rapid remodeling of mitochondrial inner membrane composition and/or area following mitotic entry. This remodeling of mitochondrial inner membrane would then have to be reverted back to previous state before mitosis had ended in order for capacitance to explain the ΔΨm dynamics in late mitosis. Our model system does not display extensive mitochondrial fission in mitosis and mitochondrial content does not change in mitosis (Fig. 1c, Supplementary Fig. 7b-d), therefore justifying our modeling with constant capacitance. Nonetheless, it is plausible that the capacitance changes to a small degree, potentially biasing our modeling results.

To enable our model to output mitochondrial ATP synthesis rate (I_{ATP}), we separated mitosis into two states, $CDK1_{off}$ and $CDK1_{on}$ states, based on the switch-like activity of CDK1. The different resistance values and the battery (E_{ETC}) can change freely between the two states. Then, in order to separate the resistance of ATP synthase (R_{ATP}), we compared the total resistance of the circuit in the presence and absence of oligomycin. This was done separate for each CDK1 state and the R_{ATP} values were used together with the ΔΨm to calculate I_{ATP} for each state (Supplementary Note 6). We circumvented dealing with model parameters other than I_{ATP} using the assumption that oligomycin only affects ATP synthase. Oligomycin is a specific inhibitor of ATP synthase[18,22] and it is not known to directly influence ETC components or mitochondrial leakage (i.e. oligomycin does not directly change R_{ETC} or R_{Leak}). The comparison between control and oligomycin treated conditions makes our model comparable to oxygen consumption-based estimation of mitochondrial ATP synthesis, such as the Seahorse Extracellular Flux Analyzer[22]. However, it is important to note that over time oligomycin treatment will influence cell physiology, as the inhibition of ATP synthase results in increased ΔΨm, changing redox metabolite levels and, eventually, in altered metabolic flux through TCA cycle. These changes can influence our

modeling result, as they also influence oxygen consumption-based ATP synthesis measurements, although the effects are considered to be small[22]. To further minimize any bias from oligomycin treatment, we have accounted for the non-ohmic $\Delta\Psi m$-dependent leakage in our model and we have minimized oligomycin treatment times during experiments (we treated cells with oligomycin only once they were close to entering mitosis). Additionally, our modeling approach quantitatively compares $\Delta\Psi m$ time-dynamics during early and late mitosis (i.e. CDK_{on} and $CDK1_{off}$ states). Therefore, any systematic bias that oligomycin may introduce to both of these states will not influence our final modeling conclusions about the relative change in ATP synthesis rate between the two states.

A similar circuit model could also have been generated using a current source instead of the voltage source coupled to a resistance. While simple and elegant, a current source does not reflect the true physical nature of the ETC, as its activity is independent of the voltage across the circuit. In contrast, the ETC's proton pumping activity depends on the $\Delta\Psi m$ due to the increasing electron leak at high membrane potentials[33,34].

Supplementary Note 4. Derivation of analytical solution to electric circuit model

Applying Kirchhoff's first law into our electric circuit (Fig. 4a), i.e. equaling the currents entering and exiting a node, we obtain:

$$I_{ETC} = I_{ATP} + I_{Leak} + I_C \tag{1}$$

Applying Ohm's law and capacitor equation to write the currents in terms of Voltage, we obtain:

$$\frac{E_{ETC} - V}{R_{ETC}} = \frac{V}{R_{ATP}} + \frac{V}{R_{Leak}} + C\frac{dV}{dt} \tag{2}$$

Rearranging equation (2), we obtain:

$$\frac{dV}{dt} + \frac{V}{R_{ATP}C} + \frac{V}{R_{Leak}C} + \frac{V}{R_{ETC}C} = \frac{E_{ETC}}{R_{ETC}C} \tag{3}$$

In equation (3), we set expressions:

$$\frac{1}{R} = \frac{1}{R_{ATP}} + \frac{1}{R_{Leak}} + \frac{1}{R_{ETC}} \tag{4}$$

$$I_S = \frac{E_{ETC}}{R_{ETC}} \tag{5}$$

Inserting expressions (4,5), into equation (3) we obtain:

$$\frac{dV}{dt} + \frac{V}{RC} = \frac{I_S}{C} \tag{6}$$

Equation (6) is a 1st order inhomogeneous ordinary differential equation with homogeneous and partial solutions:

$$V_h(t) = c\, e^{-\frac{t}{RC}} \tag{7}$$

$$V_p = I_S R \tag{8}$$

We obtain the full solution which is the sum of the homogeneous and partial solutions and includes a constant c specified by the initial conditions:

$$V(t) = c\, e^{-\frac{t}{RC}} + I_S R \tag{9}$$

At $t = t_1$ when CDK1 turns on, we have $V(t_1) = V_1$, $I_S R > V_0$ and $RC = RC_{CDK1\,on}$, thus we obtain:

$$V(t) = (V_1 - I_S R)\, e^{-\frac{t-t_1}{RC_{CDK1\,on}}} + I_S R \tag{10}$$

At $t = t_3$ when CDK1 is just turning off, we have $V(t_3) = V_3$, $I_S R = V_0$ and $RC = RC_{CDK1\,off}$, thus we obtain:

$$V(t) = (V_3 - V_0)\, e^{-\frac{t-t_3}{RC_{CDK1\,off}}} + V_0 \tag{11}$$

Given that CDK1 is on at $t = [t_1, t_2]$ and off at $t = [t_3, t_4]$ we compile equations (10,11) and write:

$$V(t) = \begin{cases} (V_1 - I_S R) e^{-\frac{t-t_1}{RC_{CDK1\,on}}} + I_S R \ , & t_1 \le t \le t_2 \\ (V_3 - V_0)\, e^{-\frac{t-t_3}{RC_{CDK1\,off}}} + V_0 \ , & t_3 \le t \le t_4 \end{cases} \tag{12}$$

Supplementary Note 5. Obtaining electrical circuit resistance values (RCs) by fitting analytical solutions to experimental data

Converting TMRE to voltage

We first normalized the experimental data of TMRE/mass signal, so that each cell has the same TMRE/mass baseline (Supplementary Fig. 11a). To identify the start ($t = t_0$) and end of mitosis ($t = t_4$) where we fit our model, we applied a smoothing filter on the normalized TMRE/mass signal (Supplementary Fig. 11b). Next, we converted the normalized TMRE/mass signal to approximate voltage (Supplementary Fig. 11c) using existing calibration curves between tetramethylrhodamidine ester fluorescence signals and membrane potentials[11]. The conversion was done using the following formula: $V(mV) = (TMRE/c_1)/c_2$, where $c_1 = 29.109$ and $c_2 = 0.0215$. For control cells we set $V_0 = -150\ mV$, which is a typical ΔΨm for many cell types [8, 9, 11, 18, 35]. For oligomycin treated cells we set $V_0 = -167.8\ mV$, which was based on the TMRE-to-voltage scaling and the measured TMRE increase in L1210 cells after a treatment with oligomycin (Supplementary Fig. 2a). Note, that while this TMRE-to-voltage conversion provides only estimates of the true ΔΨm, our final modeling results were not sensitive to the values used for TMRE-to-voltage scaling (Supplementary Note 9).

Defining fitting regions

We divided the voltage traces into regions where CDK1 is on ($t_1 \leq t \leq t_2$) and off ($t_3 \leq t \leq t_4$) (Supplementary Fig. 11d). First, we approximated the duration Δt that L1210 cells take to turn off CDK1. As CDK1 activity is lost at metaphase-anaphase transition due to the APC/C driven degradation of Cyclin B[19], we estimated the duration of this transition by quantifying the duration that L1210 cells took to degrade Geminin, another protein degraded at metaphase-anaphase transition due to APC/C activity (Supplementary Fig. 6). We then used this duration $\Delta t = 8.57 min$ to exclude the parts of mitosis where CDK1 activity is not fully on or off from our analysis. Note that the dynamics of CDK1 turning on and off have been shown to be comparable[19]. Thus, we defined the start of the region CDK1$_{on}$ ($t = t_1$) as ($t_1 = t_0 + \Delta t$). Next, we identified where CDK1 is turned off. The end of the region CDK1$_{on}$ ($t = t_2$), i.e. metaphase-anaphase transition, is near the occurrence of maximal voltage ($t = t_f$) (Fig. 1g), but the precise location of maximal voltage can be affected by noise in TMRE signal. Thus, we scanned the region around t_f to find the most optimal fitting. For every dataset for a single cell, we took eleven candidate values $t_2 = t_f - Split\, \Delta t$ (Supplementary Fig. 11d) where $Split = 1, 11, 21, \dots, 91\ or\ 99$ %. For each one of these $Split$ values, we fitted the CDK1$_{on}$ ($t = [t_1, t_2]$) and CDK1$_{off}$ regions ($t = [t_3, t_4]$) with analytical solutions from equations (10) and (11) correspondingly, excluding the Δt regions where CDK1 activity is changing. To provide the best fit to the data (Fig. 4c, Supplementary Fig. 11d), we applied the Trust Region Reflective Algorithm[36] with optimization residuals of tolerance values 10^{-6}. For the CDK1$_{on}$ region ($t = [t_1, t_2]$), we obtained optimum values for two parameters $I_S R$, $RC_{CDK1\ on}$. For the CDK1$_{off}$ region

($t = [t_3, t_4]$), we obtained the optimal value for the parameter $RC_{CDK1\,off}$. Thus, each $Split$ value yields its own $RC_{CDK1\,on}$ and $RC_{CDK1\,off}$ values with R-squared values $R^2_{CDK1\,on}$ and $R^2_{CDK1\,off}$ describing the quality of the fit. For the modeling, we utilized the Split value that provides highest combined R-squared for $RC_{CDK1\,on}$ and $RC_{CDK1\,off}$ (Supplementary Fig. 11e). The same methodology is applied to both control and oligomycin treated cells with the only difference concerning the baseline voltage as described before. Note that oligomycin had little effect on the mitotic progression (Fig. 3c, d), thus allowing us to use the same Δt as for control. Thus, for both control and oligomycin treated cells we obtained the time constant $RC_{CDK1\,on}$ and $RC_{CDK1\,off}$ values (Fig. 4d) by fitting or model solution to the regions $CDK1_{on}$ and $CDK1_{off}$, respectively.

Data exclusion from the model

It is important to note that we kept datasets of single cells when they satisfy two criteria. First, our model needs to yield a physiologically relevant parameters, in particular the $I_S R$ value (maximum projected voltage of hyperpolarization). We excluded any control and oligomycin fits that yielded $I_S R$ value above -203mV , as experiments with isolated mitochondria have indicated that mitochondrial membrane potential cannot increase beyond values of approximately -225 mV due to the non-ohmic proton conductance[29,37-39] and live cell experiments have suggested lower ΔΨm limits[22,32]. Second, our modeling approach requires the TMRE signal (voltage) to establish a clear new baseline before the abscission of daughter cells to successfully derive RC values from exponential curve fitting (equation (11)). Therefore, we excluded cells that divided before a new baseline was established. In total these exclusion criteria resulted in using 85 cells of the measured 124 control cells and 32 out of the 38 measured oligomycin cells.

Supplementary Note 6. Calculating ATP current (I_{ATP}) from RC values

Approach for calculating I_{ATP} currents

To calculate the current I_{ATP} we used Ohm's law using the voltage and the $R_{ATP}C$ values for each case of $CDK1\ on$ and $CDK1\ off$:

$$\frac{I_{ATP,CDK1\,off}}{C} = V\left(\frac{1}{R_{ATP}C}\right)_{CDK1\,off} \tag{13}$$

$$\frac{I_{ATP,CDK1\,on}}{C} = V\left(\frac{1}{R_{ATP}C}\right)_{CDK1\,on} \tag{14}$$

Note that we calculated the currents per capacitance C which we assumed to be constant as justified above (Supplementary Note 3). We used the voltage V values converted from the experimentally measured TMRE. To calculate $R_{ATP}C$ for each case of $CDK1\ on$ and $CDK1\ off$ we used the mean values of the time constants $RC_{CDK1\,on}$ and $RC_{CDK1\,off}$. Note that $RC_{CDK1\,on}$ and $RC_{CDK1\,off}$ originate from single-cell experiments, but the derivation of $R_{ATP}C$ requires comparisons between control and oligomycin conditions. Therefore, we calculated two mean values $R_{ATP}C$, one for $CDK1\ on$ and one for $CDK1\ off$. We then applied these values to all control cell voltage traces in order to calculate I_{ATP} based on equations (13,14), while taking in to account the non-ohmic scaling of leakage with increasing voltage. Below, for simplicity in writing for the rest of this section, we omitted the mention of subscripts $CDK1\ on$ and $CDK1\ off$. Moreover, we referred to mean value as RC with superscripts of C for control, O for oligomycin treated. In addition, we used subscripts of $ATP, Leak, ETC$ for different components of the electrical circuit, consistently with equations (2-5) and main text (Fig. 4a).

I_{ATP} for CDK1$_{off}$

For control cells, we apply equation (4):

$$\left(\frac{1}{RC}\right)^{C} = \left(\frac{1}{R_{ATP}C}\right)^{C} + \left(\frac{1}{R_{Leak}C}\right)^{C} + \left(\frac{1}{R_{ETC}C}\right)^{C} \tag{15}$$

For oligomycin treated cells, since ATP synthase in inhibited by oligomycin, we can neglect term R_{ATP}. Similarly to control cells, we write

$$\left(\frac{1}{RC}\right)^{O} = \underbrace{\left(\frac{1}{R_{ATP}C}\right)^{O}}_{=0} + \left(\frac{1}{R_{Leak}C}\right)^{O} + \left(\frac{1}{R_{ETC}C}\right)^{O} \tag{16}$$

Our goal is to calculate $(1/R_{ATP}C)^C$ from equation (15). Since the left-hand terms of equations (14,15) are specified as the mean values from experimental data, we need an expression for $(1/R_{Leak}C)^C$ and $(1/R_{Leak}C)^O$ terms. As the leakage current (I_{Leak}) is known to be voltage-dependent with a non-ohmic scaling $I_{Leak} \propto e^{\gamma V}$ [23,32,33], the resistance term R_{Leak} is voltage-dependent as well. Therefore, we write a function that is voltage-dependent $l(V) = (1/R_{Leak}C)$. Consequently, the term $(1/R_{ATP}C)$ is voltage-dependent and we write $a(V) = (1/R_{ATP}C)$. As justified above (Supplementary Note 3), we assume that that the term $(1/R_{ETC}C)$ for electron transport chain is the same for both control and oligomycin treated cells, that is $(1/R_{ETC}C)^C = (1/R_{ETC}C)^O = (1/R_{ETC}C)$. In the case of control in equation (16), we can write:

$$\left(\frac{1}{RC}\right)^C = a(V) + l(V) + \left(\frac{1}{R_{ETC}C}\right) \tag{17}$$

However, in case of oligomycin in equation (17) we can treat the term $l(V) = (1/R_{Leak}C)^O$ as approximately constant at a representative median voltage $\bar{V}_o$ (median voltage hyperpolarization with oligomycin), write $l(\bar{V}_o\,) = (1/R_{Leak}C)^O$and obtain:

$$\left(\frac{1}{RC}\right)^O = l(\bar{V}_o\,) + \left(\frac{1}{R_{ETC}C}\right) \tag{18}$$

Subtracting equation (18) from equation (17), we obtain:

$$\left(\frac{1}{RC}\right)^C - \left(\frac{1}{RC}\right)^O = a(V) + l(V) - l(\bar{V}_o\,) \tag{19}$$

To extract an expression for $a(V)$, we need an expression for leakage term $l(V)$. To achieve this, we use previously reported scaling between leakage current and voltage [32] as follows: $I_{Leak} \propto e^{\gamma V}$, where $\gamma = 0.0122$. From Ohm's law, $I_{Leak} = V/R_{Leak}$, we can write $R_{Leak}C \propto Ve^{-\gamma V}$. Therefore, we express the function $l(V)$ using l_o as a proportionality constant:

$$l(V) = l_o e^{\gamma V}/V \tag{20}$$

Inserting equation (20) into equation (19), we obtain:

$$\left(\frac{1}{RC}\right)^C - \left(\frac{1}{RC}\right)^O = a(V) + l_o e^{\gamma V}/V - l_o e^{\gamma \bar{V}_o}/\bar{V}_o \tag{21}$$

To specify the proportionality constant l_o, we set $V = V_{G2}$, which is the membrane potential in G2 phase (prior to the mitotic mitochondrial hyperpolarization). We then quantified the ATP synthesis to leakage ratio (I_{ATP}/I_{Leak}) by measuring the oligomycin sensitive and insensitive proportions of mitochondrial oxygen consumption using a Seahorse Extracellular Flux Analyzer in L1210 cells (Supplementary Fig. 8a, c). Based on these measurements we set $I_{ATP}/I_{Leak}|_{V=V_{G2}} = \beta$ where $\beta = 4.88$. Using Ohm's law, we write $a(V_{G2})/l(V_{G2}) = \beta$. We then set $V = V_{G2}$ (-150 mV) in equation (21) and obtain:

$$\left(\frac{1}{RC}\right)^C - \left(\frac{1}{RC}\right)^O = (1+\beta)l_o e^{\gamma V_{G2}}/V_{G2} - l_o e^{\gamma \bar{V}_o}/\bar{V}_o \tag{22}$$

We can therefore solve equation (22) with respect to l_o and obtain:

$$l_o = \frac{\left(\frac{1}{RC}\right)^C - \left(\frac{1}{RC}\right)^O}{\frac{(1+\beta)e^{\gamma V_{G2}}}{V_{G2}} - \frac{e^{\gamma \bar{V}_o}}{\bar{V}_o}} \tag{23}$$

Inserting equation (23) into equation (19), we obtain an expression for $(1/R_{ATP}C)^C_{CDK1\,off}$. Note that now we have added the subscript 'CDK1 off' for clarity:

$$\begin{aligned}\left(\frac{1}{R_{ATP}C}\right)^C_{CDK1\,off} \\ &= \left(\frac{1}{RC}\right)^C_{CDK1\,off} - \left(\frac{1}{RC}\right)^O_{CDK1\,off} \\ &- \left(\frac{\left(\frac{1}{RC}\right)^C_{CDK1\,off} - \left(\frac{1}{RC}\right)^O_{CDK1\,off}}{\frac{(1+\beta)e^{\gamma V_{G2}}}{V_{G2}} - \frac{e^{\gamma \bar{V}_o}}{\bar{V}_o}}\right)\left(\frac{e^{\gamma V}}{V} - \frac{e^{\gamma \bar{V}_o}}{\bar{V}_o}\right)\end{aligned} \tag{24}$$

I_{ATP} for $CDK1_{on}$

In the case of $CDK1_{on}$ we apply the same methodology for control and oligomycin cells as in the case of $CDK1_{off}$, as shown in equations (15-23). We set the leakage terms $(1/R_{Leak}C)$ in $CDK1_{on}$ and $CDK1_{off}$ to be connected through equation (25), where c is a constant:

$$l_{CDK1\,on}(V) = l(V) + c \tag{25}$$

For $CDK1_{on}$ we can therefore write:

$$\left(\frac{1}{R_{ATP}C}\right)^{C}_{CDK1\,on} = \left(\frac{1}{RC}\right)^{C}_{CDK1\,on} - \left(\frac{1}{RC}\right)^{O}_{CDK1\,on} - \left(\frac{\left(\frac{1}{RC}\right)^{C}_{CDK1\,off} - \left(\frac{1}{RC}\right)^{O}_{CDK1\,off}}{\frac{(1+\beta)e^{\gamma V_{G2}}}{V_{G2}} - \frac{e^{\gamma \bar{V}_o}}{\bar{V}_o}}\right)\left(\frac{e^{\gamma V}}{V} - \frac{e^{\gamma \bar{V}_o}}{\bar{V}_o}\right) \qquad (26)$$

Having specified the values for $R_{ATP}C$ for both $CDK1_{on}$ and $CDK1_{off}$, we can calculate the ATP currents using equations (13,14). For the Δt regions, where CDK1 transitions from off to on ($t = [t_0, t_1]$) and vice versa ($t = [t_2, t_3]$), we linearly interpolated the ATP currents between the transition points.

To extract mean and standard deviation of ATP currents (I_{ATP}) for each cell, we utilized the population average $R_{ATP}C$ values and the TMRE measurement-based estimates of voltage. We first linearly interpolated the current of each cell obtained from equations (13) and (14) to have 1,000 data points around mitosis (starting from 2 h before and ending at 1.5 h after metaphase-anaphase transition ($t = t_f$)). Then, we aligned each interpolated current trace at the metaphase-anaphase transition and calculated mean ± standard deviation at each time point that was linearly interpolated.

General implications of RC values

Our calculations revealed that $R_{ATP}C$ is higher during $CDK1\ on$ state than during $CDK1\ off$ state, 0.7 ± 0.2 and 0.27 ± 0.01 h respectively (mean ± SEM). These values are the mean $R_{ATP}C$ values over a voltage range of -150 mV to -200mV (Supplementary Fig. 12c). Our data also revealed that in control cells $RC_{CDK1\,on}/RC_{CDK1\,off} = 1.4 \pm 0.1$ (mean ± SEM), which is statistically different from the value 1.0 ($p = 1.8 * 10^{-4}$, unpaired, two-tailed Student's t-test, n=85). Thus, the overall time constant RC increases in $CDK1_{on}$ state. Note that the overall time constant RC is determined, in addition to $R_{ATP}C$, by the terms $R_{ETC}C$ and $R_{Leak}C$ based on equation (15). We therefore questioned if $R_{ETC}C$ and/or $R_{Leak}C$ may also change during $CDK1_{on}$ state. To test this hypothesis, we set $R_{ETC}C$ and $R_{Leak}C$ to stay constant between $CDK1_{on}$ and $CDK1_{off}$ states, with only $R_{ATP}C$ becoming higher during $CDK1_{on}$ state, as shown above. This hypothetical ratio $RC_{CDK1\,on}/RC_{CDK1\,off} = 1.8 \pm 0.7$ is significantly different from our measured ratio of 1.4 ± 0.1 (mean ± SEM) ($p = 8 * 10^{-4}$, unpaired, two-tailed Student's t-test, n=85). Therefore, our model predicts that $R_{ETC}C$ and/or $R_{Leak}C$ decrease during $CDK1_{on}$.

This prediction of our model that $R_{ETC}C$ and/or $R_{Leak}C$ decrease (i.e. ETC activity and/or leakage increase) during mitosis ($CDK1_{on}$ state) is consistent with literature. It has been reported that ETC activity increases during mitosis[40-42], and our observation that TMRE increases upon

mitotic entry in oligomycin treated cells also implies increased ETC activity during mitosis (Fig. 3a, b). However, when we synchronized cells to mitosis and measured their oxygen consumption rate in comparison to G2 arrested cells, we did not observe increased oxygen consumption (Supplementary Fig. 8d, e). This likely reflects an artefact of cell cycle synchronization, as mitotic arrests are known to result in inhibition of cell growth and degradation of mitochondria[3,41]. In addition, the mitotically arrested cells displayed higher mitochondrial leakage than G2 arrested cells (Supplementary Fig. 8d, e), implying that $R_{Leak}C$ is also decreased during mitosis. As leakage also increases with increased $\Delta\Psi m$[32], mitotic cells should display increased mitochondrial proton leakage. Mitochondrial proton leakage is the main heat source within cells[43, 44] and, consistently, cells in early mitosis have been shown to produce more heat[45]. Thus, while our model cannot quantify the extent to which $R_{ETC}C$ and $R_{Leak}C$ change, it seems likely that both values decrease during mitosis ($RC_{CDK1\,on}$ state) and thus mitotic cells would display increased I_{ETC} and I_{Leak}.

Supplementary Note 7. Calculating ratios and integral of current I_{ATP}/C

As the key conclusion of our model, we calculate the extent of ATP synthesis rate change between G2 and mitotic cells, which we call $r_{I_{ATP}}$. To do this, we obtain the ratio between the currents I_{ATP}/C from equations (13,14):

$$r_{I_{ATP}} = \frac{I_{ATP,CDK1\,on}}{I_{ATP,CDK1\,off}} \tag{27}$$

To calculate $r_{I_{ATP}}$ from equation (27), we used the minimum value of mean $I_{ATP,CDK1\,on}$ and the baseline (G2) value of mean $I_{ATP,CDK1\,off}$ of control cells.

We also integrated the equations (13,14) using the trapezoidal rule method over discrete points (t_i , I_{ATP}/C_i) to obtain the total charge transported across the inner mitochondrial membrane through ATP synthase, i.e. the amount of ATP synthesized. Here, I_{ATP}/C_i can refer either to $CDK1_{on}$ or $CDK1_{off}$:

$$Q_{I_{ATP}} = \sum_{i=2}^{N} \frac{I_{ATP}/C_i + I_{ATP}/C_{i-1}}{2} \Delta t = \left(\frac{I_{ATP}/C_0}{2} + \sum_{i=2}^{N-1} I_{ATP}/C_i + \frac{I_{ATP}/C_N}{2} \right) \Delta t \tag{28}$$

Note that in equation (28) we did the integration over the mean ATP synthesis rate we derived from control sets, which we had interpolated (Supplementary Note 6). Due to the interpolation in time t, the time step was constant, $\Delta t = t_{i+1} - t_i = constant$.

The integration was done for ATP currents in early mitosis (spanning 30 min from G2/M transition to metaphase-anaphase transition) and for anaphase (spanning 15 minutes from metaphase-anaphase transition to the approximate end of anaphase)[3,5]. As a reference, these values were compared to a hypothetical situation where the ATP synthesis rate remained at G2 levels.

Supplementary Note 8. Error analysis

We calculate the error in term $(R_{ATP}C)$ of equations (24 and 26) for CDK1$_{on}$ and CDK1$_{off}$ respectively, by accounting for the error in the estimation of $(RC)^C$, $(RC)^O$, where superscript C refers to control and O to oligomycin data. We did not include error of voltage V in these calculations as the extent to which our TMRE-to-voltage conversion factors and our voltage dependent leakage factors influence the voltage values is not known and thus we cannot directly estimate their contribution to the error. Instead, we carried out sensitivity analyses on the TMRE-to-voltage conversion (Supplementary Fig. 13) and the voltage-dependent leakage (Supplementary Fig. 15, Supplementary Note 9). Note also that our the TMRE measurements of mitotic cells is typically below 2 % (Supplementary Fig. 1d). Below, we do the error analysis for CDK1$_{on}$ and CDK1$_{off}$ separately.

Case CDK1$_{off}$

To estimate the error s_f of a function f which depends on two variables x, y with errors s_x, s_y we use equation (29), assuming that x, y are independent, uncorrelated, variables:

$$s_f = \sqrt{\left(\frac{\partial f}{\partial x} s_x\right)^2 + \left(\frac{\partial f}{\partial y} s_y\right)^2} \tag{29}$$

For simplicity in writing of equation (24) we can use the following symbols, where $\delta(RC)^C_{CDK1\,off}$ and $\delta(RC)^O_{CDK1\,off}$ stand for standard errors for $(RC)^C_{CDK1\,off}$ and $(RC)^O_{CDK1\,off}$ respectively:

$$f_{off} = (R_{ATP}C)^C_{CDK1\,off} \tag{30}$$
$$x = (RC)^C_{CDK1\,off} \tag{31}$$
$$y = (RC)^O_{CDK1\,off} \tag{32}$$
$$s_x = \delta(RC)^C_{CDK1\,off} \tag{33}$$
$$s_y = \delta(RC)^O_{CDK1\,off} \tag{34}$$

Note that from our datasets we obtained $s_x/x = 4\%$ and $s_y/y = 8\%$ (Fig. 4d), which means that the standard errors are relatively small with respect to the mean values, thus justifying the use of equation (29).

As stated above, in our calculations the rest of the terms carry no error. Thus, we can group the terms together under a variable G:

$$G = \left(\frac{1}{\frac{(1+\beta)e^{\gamma V_{G2}}}{V_{G2}} - \frac{e^{\gamma \bar{V}_o}}{\bar{V}_o}} \right) \left(\frac{e^{\gamma V}}{V} - \frac{e^{\gamma \bar{V}_o}}{\bar{V}_o} \right) \tag{35}$$

Therefore equation (24) can be written as:

$$f_{off} = \frac{1}{\frac{1-G}{x} + \frac{G-1}{y}} \tag{36}$$

We take the derivative of equation (34) with respect to x and y and write this in compact form since each derivative is proportional to f_{off}^2:

$$\frac{\partial f_{off}}{\partial x} = -\frac{(1-G)f_{off}^2}{x^2} \tag{37}$$

$$\frac{\partial f_{off}}{\partial y} = -\frac{(G-1)f_{off}^2}{y^2} \tag{38}$$

Therefore, we estimate the error $\delta(R_{ATP}C)_{CDK1\,off}^{C}$ combining equations (29-38):

$$\delta(R_{ATP}C)_{CDK1\,off}^{C} = \left[(R_{ATP}C)_{CDK1\,off}^{C}\right]^2 \mathrm{x}$$
$$|\mathrm{G}-1| \sqrt{\left(\frac{\delta(RC)_{CDK1\,off}^{C}}{[(RC)_{CDK1\,off}^{C}]^2}\right)^2 + \left(\frac{\delta(RC)_{CDK1\,off}^{O}}{[(RC)_{CDK1\,off}^{O}]^2}\right)^2} \tag{39}$$

From equation (39) it is clear that the error $\delta(R_{ATP}C)_{CDK1\,off}^{C}$ depends on voltage V included in the expression of G from equation (35).

Case CDK1$_{on}$

For simplicity with symbols, we set:

$$f_{on} = (R_{ATP}C)_{CDK1\,on}^{C} \tag{40}$$
$$z = (RC)_{CDK1\,on}^{C} \tag{41}$$
$$w = (RC)_{CDK1\,on}^{O} \tag{42}$$
$$s_z = \delta(RC)_{CDK1\,on}^{C} \tag{43}$$
$$s_w = \delta(RC)_{CDK1\,on}^{O} \tag{44}$$

Note that in this case we also have $s_z/z = 8\%$ and $s_w/y = 12\%$ (Fig. 4d) which means that the standard errors are relatively small with respect to the mean values, thus justifying the use of an equation similar to (29) but with four terms.

$$s_f = \sqrt{\left(\frac{\partial f}{\partial x}s_x\right)^2 + \left(\frac{\partial f}{\partial y}s_y\right)^2 + \left(\frac{\partial f}{\partial z}s_z\right)^2 + \left(\frac{\partial f}{\partial w}s_w\right)^2} \quad (45)$$

Similar to the case CDK1$_{on}$, we apply equations (31-34, 40-45) to equation (26) and obtain:

$$f_{on} = \frac{1}{\frac{1}{z} - \frac{1}{w} - \frac{G}{x} + \frac{G}{y}} \quad (46)$$

We take the derivatives of equation (46) with respect to z, w, x, y and write this in compact form since each derivative is proportional to f_{on}^2:

$$\frac{\partial f_{on}}{\partial x} = \frac{G f_{on}^2}{x^2} \quad (47)$$

$$\frac{\partial f_{on}}{\partial y} = -\frac{G f_{on}^2}{y^2} \quad (48)$$

$$\frac{\partial f_{on}}{\partial z} = -\frac{f_{on}^2}{z^2} \quad (49)$$

$$\frac{\partial f_{on}}{\partial w} = \frac{f_{on}^2}{w^2} \quad (50)$$

Therefore, we estimate the error $\delta(R_{ATP}C)^C_{CDK1\,on}$ by combining equations (40-50):

$$\delta(R_{ATP}C)^C_{CDK1\,on} = [(R_{ATP}C)^C_{CDK1\,on}]^2 \text{ x}$$
$$\sqrt{\left(\frac{G\delta(RC)^C_{CDK1\,off}}{[(RC)^C_{CDK1\,off}]^2}\right)^2 + \left(\frac{G\delta(RC)^O_{CDK1\,off}}{[(RC)^O_{CDK1\,off}]^2}\right)^2 + \left(\frac{\delta(RC)^C_{CDK1\,on}}{[(RC)^C_{CDK1\,on}]^2}\right)^2 + \left(\frac{\delta(RC)^O_{CDK1\,c}}{[(RC)^O_{CDK1\,on}} \right.} \quad (51)$$

Similar to equation (39), the error $\delta(R_{ATP}C)^C_{CDK1\,on}$ depends on voltage V included in the expression of G from equation (35).

Calculating errors for currents I_{ATP}/C, its ratios and integrals

Using expressions (39, 51) for the errors for $(R_{ATP}C)^C$ we calculate the error in the current I_{ATP}/C from equations (13,14). Applying the same methodology we used for deriving equations (39,51), we obtain:

$$\delta\left(\frac{I_{ATP,CDK1\,off}}{C}\right) = \frac{I_{ATP,CDK1\,off}}{C}\left|\frac{\delta(R_{ATP}C)^C_{CDK1\,off}}{(R_{ATP}C)^C_{CDK1\,off}}\right| \tag{52}$$

$$\delta\left(\frac{I_{ATP,CDK1\,on}}{C}\right) = \frac{I_{ATP,CDK1\,on}}{C}\left|\frac{\delta(R_{ATP}C)^C_{CDK1\,on}}{(R_{ATP}C)^C_{CDK1\,on}}\right| \tag{53}$$

We calculate the error for $r_{I_{ATP}}$ in a similar fashion as before:

$$\delta r_{I_{ATP}} = \frac{R_{ATP,CDK1\,off}}{R_{ATP,CDK1\,on}}\sqrt{\left(\frac{\delta(R_{ATP}C)^C_{CDK1\,off}}{(R_{ATP}C)^C_{CDK1\,off}}\right)^2 + \left(\frac{\delta(R_{ATP}C)^C_{CDK1\,on}}{(R_{ATP}C)^C_{CDK1\,on}}\right)^2} \tag{54}$$

Using a formula of partial derivatives for the errors similar to equations (29,45), we calculate the error of the integral, $\delta Q_{I_{ATP}}$ as:

$$\delta Q_{I_{ATP}} = \Delta t\sqrt{\left[\delta\left(\frac{I_{ATP}/C_0}{2}\right)\right]^2 + \sum_{i=2}^{N-1}[\delta(I_{ATP}/C_i)]^2 + \left[\delta\left(\frac{I_{ATP}/C_N}{2}\right)\right]^2} \tag{55}$$

Supplementary Note 9. Sensitivity analysis for model

First, we carried out a sensitivity analysis on the selection criterion for the best $Split$ value (Supplementary Fig. 11e). In addition to selecting the $Split$ value based on maximization of R-squared, we also selected the $Split$ value based on maximization of RC value confidence (Supplementary Fig. 12b). We defined the RC value confidence as $|RC_{max} - RC_{min}|/RC$, where RC_{max}, RC_{min} represent the maximum and minimum value of RC with 95% confidence. We observed little difference between the two selection criteria (Supplementary Fig. 12b, mean values of RC) and thus decided to use the maximization of R-squared as a selection criterion for the $Split$ value.

After establishing the maximization of R-squared as a selection criterion, we carried out sensitivity analyses on both our own model parameters, as well as the parameters we obtained from literature. In these analyses we tested the variability of our basic output from our model: the ratio of ATP synthesis between mitosis and G2, $r_{I_{ATP}} = \frac{I_{ATP,CDK1\,on}}{I_{ATP,CDK1\,off}}$. The value we calculated using the model was $r_{I_{ATP}} = 46\ \% \ \pm\ 11\ \%$ (mean ± SEM), and was obtained using the following parameters:

1) Baseline voltage $V_o = -150\ mV$ (Supplementary Note 5)
2) TMRE-to-ΔΨm conversion factor $c_2 = 0.0215$ (Supplementary Note 5)
3) For ΔΨm-dependent leakage $\beta = 4.88, \gamma = 0.0122$ (Supplementary Note 6)
4) Start angle for data fitting ($\theta_{start} = 15\ degrees$) (Supplementary Note 5, Supplementary Fig. 11b)
5) The duration of CDK1 turning on and off ($\Delta t = 8.57\ min$) (Supplementary Note 5, Supplementary Fig. 6).

First, we calculated $r_{I_{ATP}}$ when changing the parameters of TMRE-to-ΔΨm conversion factor ($c_2 = 0.0185, 0.02, 0.0215, 0.0230, 0.0245$) and the assumed baseline voltage ($\Delta\Psi\text{m} = -140, -151, \ldots, -160\ mV$) while keeping the rest of the parameters fixed. Over the range of these parameters $r_{I_{ATP}}$ was fluctuating within the range $35 - 50\%$ (Supplementary Fig. 13a), indicating that our model is not sensitive to the values used for TMRE-to-voltage conversion. We also calculated the relative error (Supplementary Fig. 13b) in the estimation of $r_{I_{ATP}}$ based on the equation:

$$R.E.\,\delta r_{I_{ATP}} = \frac{\delta r_{I_{ATP}}}{r_{I_{ATP}}} \tag{56}$$

We observed that $R.E.\,\delta r_{I_{ATP}}$ is kept below 30% (Supplementary Fig. 13c) throughout this range. In other words, the extent of our uncertainty in the estimation of $r_{I_{ATP}}$ is relatively small. Moreover, we calculated the data inclusion based on our criteria, as well as fitting quality (R-squared of the

fits), and observed that throughout the range, over 95% of data were preserved (Supplementary Fig. 13b) and the mean quality of the fits stays consistently high (average $R^2 > 0.94$) (Supplementary Fig. 13d).

Second, we calculated $r_{I_{ATP}}$ when changing the parameters used to define fitting regions. We changed the parameters used for to define where fitting starts ($\theta_{start} = 5{,}10{,}15{,}20\ degrees$) and the duration of CDK1 on/off transitions ($\Delta t = 6{,}7{,}8.57{,}9{,}10\ min$), while keeping the rest of the parameters fixed. Over the range of these parameters $r_{I_{ATP}}$ fluctuated within the range $15 -$ 50% (Supplementary Fig. 14a). The $r_{I_{ATP}}$ change was more pronounced when θ_{start} changed. However, we note that a lower starting angle ($\theta_{start} = 5 - 10\ degrees$) is associated with more noise in the data leading to a higher $R.E.\delta r_{I_{ATP}}$ (Supplementary Fig. 14c). Notably, the $R.E.\delta r_{I_{ATP}}$ was minimized at our reference values. In addition, more single-cell data sets were excluded when θ_{start} and Δt decreased (Supplementary Fig. 14b) (see Supplementary Note 5 for data exclusion criteria), although the R-squared of fits remained high (Supplementary Fig. 14d).

Third, we calculated $r_{I_{ATP}}$ for multiple pairs of the values (β, γ), which define the voltage-dependent leakage in our model. For each pair from combining values ($\beta = 4, 4.88, 6$) and ($\gamma = 0, 0.005, 0.0122{,}0.015, 0.02, 0.03$), we computed $r_{I_{ATP}}$ over a sweep of the parameters for TMRE-to-ΔΨm conversion factor ($c_2 = 0.0185, 0.02{,}0.0215, 0.0230, 0.0245$) and baseline voltage ($\Delta\Psi m = 140, 151, \ldots, 160$). Over these ranges, we observed that $r_{I_{ATP}}$ fluctuated within the range $35 - 55\%$ (Supplementary Fig. 15). Note that at $\gamma = 0$ the leakage current has an ohmic scaling with ΔΨm. Thus, our sensitivity analysis overall indicates that the results of our model have little sensitivity to the assumed and/or literature-derived values. We conclude that the result of our model, i.e. that L1210 cells decrease their mitochondrial ATP synthesis approximately 55% during early mitosis when compared to G2 levels, is reflecting the underlying biological changes in mitosis and not a modeling artefact.

Supplementary Figures

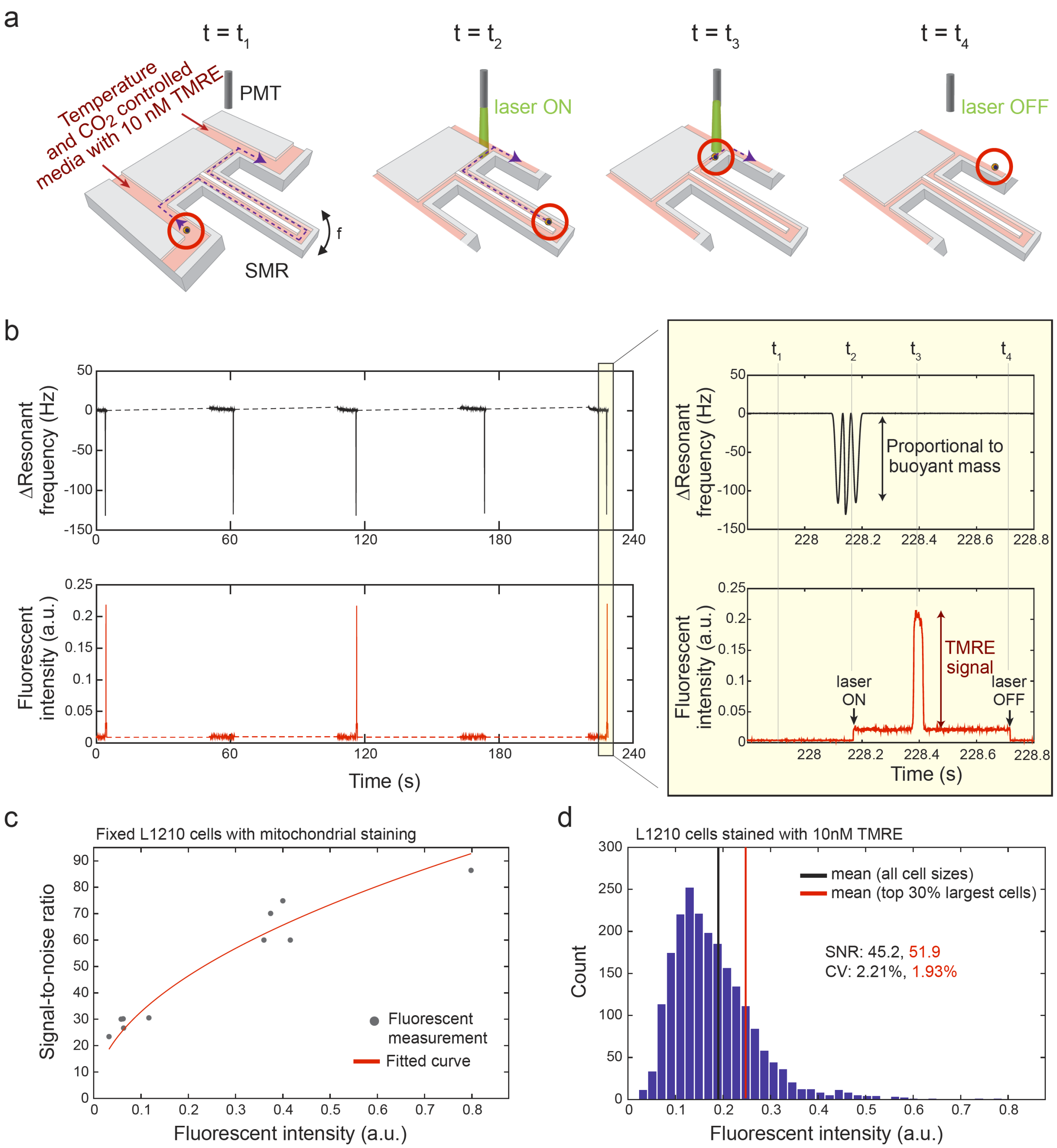

Supplementary Fig. 1 Fluorescence detection system coupled to suspended microchannel resonator (SMR) allows precise mass-normalized TMRE signal quantifications.

a Schematic of the SMR measurement setup. A cell (highlighted by red circles) is grown in environmentally controlled conditions in normal cell culture media within the SMR[1-3]. Approximately every 1 min, the cell is flown through a microchannel inside the SMR cantilever (t = t_1, the trajectory of the cell is shown with purple dotted lines). This results in a change in the cantilever's resonance frequency that is proportional to the buoyant mass of the cell (t = t_2). The

change in resonance frequency turns on a laser that is focused to the microchannel outside the SMR cantilever. As the cell passes through the laser excitation ($t = t_3$), the emission signal from the cell is captured using photomultiplier tubes (PMTs). Then, the laser turns off and the fluid flow is stopped for approximately 1 minute, after which the cell is flown back to the other side.

b Example raw data of SMR resonant frequency and PMT based fluorescent detection during five consecutive buoyant mass measurements (*left*) along with a zoom-in of a single measurement of buoyant mass and TMRE signal (*right*). When a cell goes through the SMR its buoyant mass (double black arrow; height of side resonant frequency peak) acts as a trigger to turn on the laser. Approximately 200 ms later the cell flows through the laser illumination where the TMRE signal (red double arrow; height of the PMT voltage spike) is quantified using the PMTs. Approximately 200 ms later the laser turns off to minimize phototoxicity from scattered light. In data analysis each TMRE signal intensity is normalized to the corresponding buoyant mass measurement to account for cell size-dependent differences.

c Quantification of fluorescent error for mitochondrial staining. Mitochondria in L1210 cells were stained with 100 nM or 333 nM MitoTracker Red CMXRos, the cells were fixed and then each cell was repeatedly measured to quantify fluorescence errors. Signal-to-noise ratio is displayed for each cell (gray dots, N=10 cells, each repeatedly measured >50 times). Estimated signal-to-noise ratio (SNR) assuming perfect shot noise (i.e., SNR $\propto \sqrt{n}$, where n is the average number of photons detected on PMT per unit time) is displayed in red.

d Estimated SNR and Coefficient of Variation (CV) of typical TMRE measurement. Fluorescent Intensity of L1210 cells treated with 10nM TMRE and their distribution is shown in the blue histogram. Estimated SNR and CV of the TMRE measurements is shown for average cells in a population (black) and for top 30% largest cells in a population, which reflect mostly G2 and mitotic cells (red). SNR and CV were calculated using the fitted curve displayed in panel **(c)**.

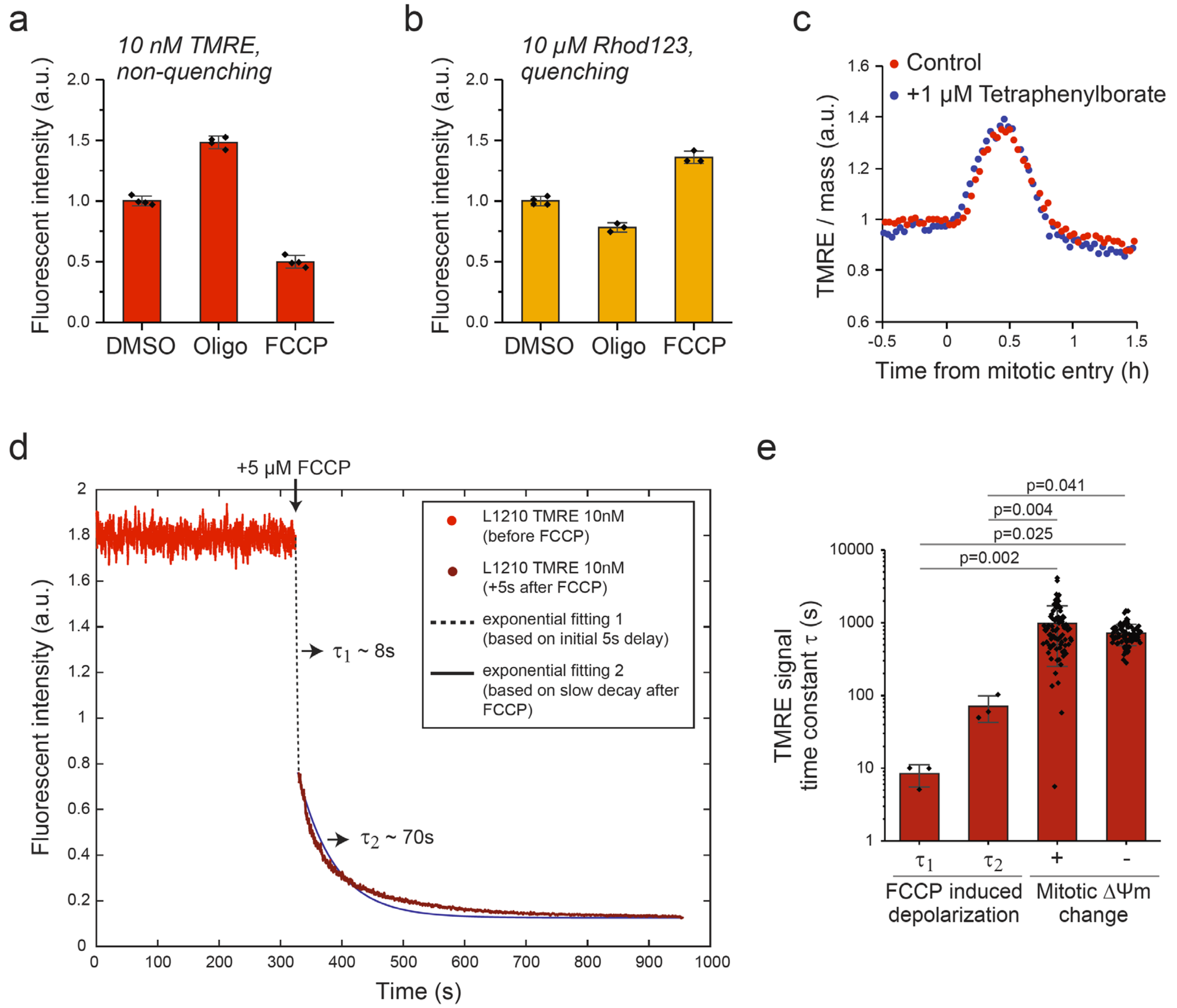

Supplementary Fig. 2 Detection of the mitochondrial hyperpolarization at the end of the cell cycle is not limited by TMRE diffusion kinetics when TMRE is used in non-quenching mode.
a Population average TMRE fluorescence intensity for L1210 cells after 30 min treatment 1 µM oligomycin or 1 µM FCCP. The directionality of the fluorescence change indicates that TMRE is acting in a non-quenching mode. Data represents mean ± SD (N=4 independent cultures).
b Population average Rhod123 fluorescence intensity for L1210 cells after 30 min treatment 1 µM oligomycin or 1 µM FCCP. The directionality of the fluorescence change indicates that Rhod123 is acting in a quenching mode. Note that Rhod123 was removed from the culture media prior to the addition of the chemicals. Data represents mean ± SD (N=4 independent cultures for DMSO treatment, N=3 independent cultures for Oligomycin and FCCP treatments).
c Mass-normalized TMRE (10 nM) traces for a L1210 cell around cell division in the presence and absence of 1 µM Tetraphenylborate, which promotes the diffusion of TMRE through membranes[7,8].

d Representative data used for quantifications of TMRE diffusion kinetics (N=3 independent experiments). L1210 cells were stained with 10 nM TMRE and the average population TMRE intensity was sampled using flow cytometer before (red) and after (orange) treatment with the protonophore FCCP (5 μM). The time constants were extracted by i) fitting an exponential decrease between the last data point prior to FCCP addition and the first data point after FCCP addition (τ_1), or by ii) fitting an exponential decrease to the data after FCCP addition (τ_2).
e Quantifications of TMRE signal time constants during FCCP induced depolarization (τ_1 and τ_2 from panel **(d)**, N=3 independent experiments) and during the TMRE increase (+) and decrease (-) in mitosis (N=85 cells from 40 independent experiments). Note that the rate at which TMRE signal changes in mitosis is at least an order of magnitude slower than what TMRE signal can change. Data represents mean ± SD. Statistical significance was assessed using one-way ANOVA followed by Sidakholm test. Source data are provided as a Source Data file.

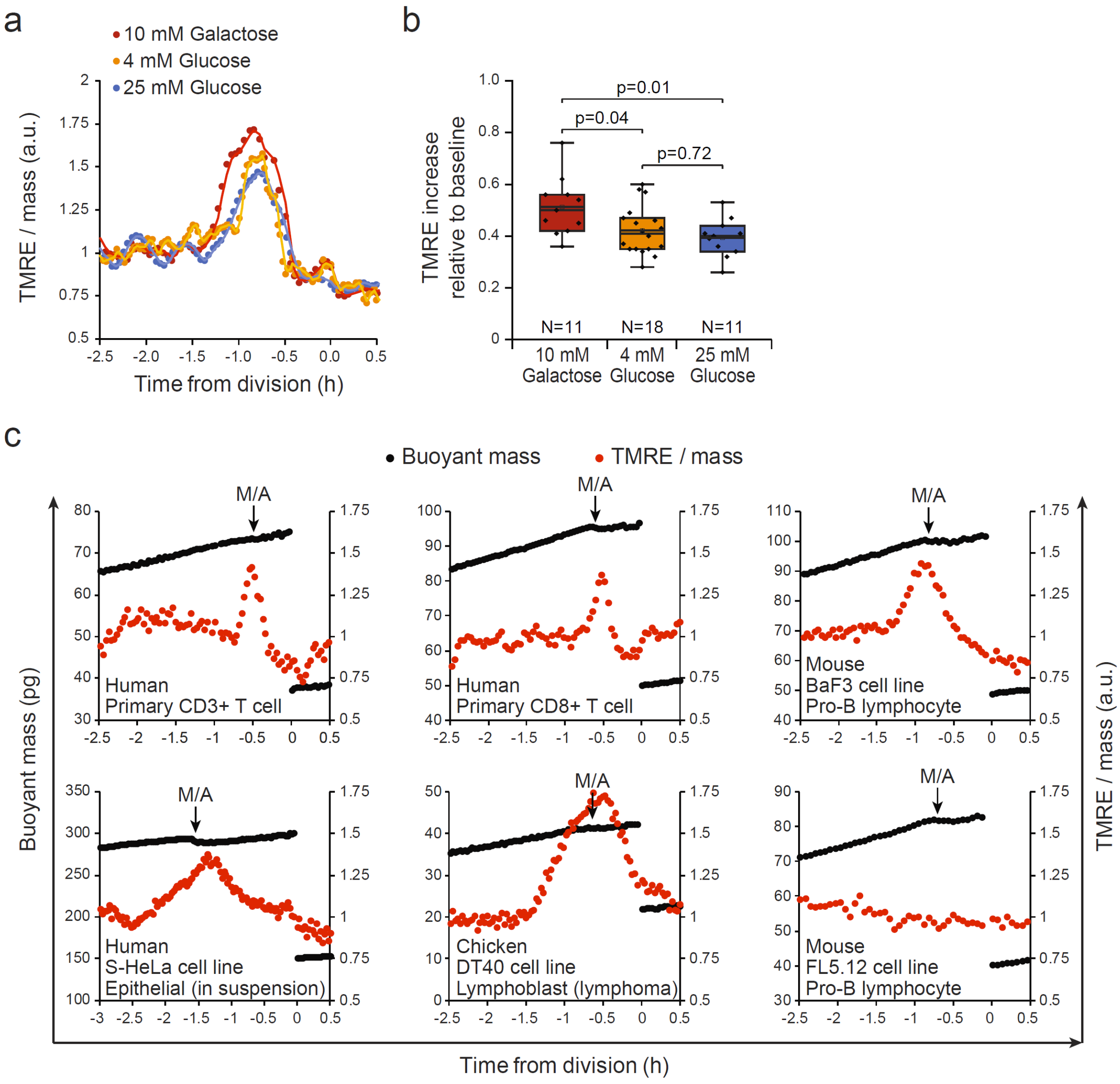

Supplementary Fig. 3 Mitochondrial hyperpolarization takes place at the end of cell cycle in various animal cell types and under different glucose concentrations.

a Mass-normalized TMRE traces for L1210 cells around cell division under indicated nutrient conditions. Galactose grown cells did not have glucose in the culture media.

b Quantifications of L1210 cell TMRE increase in mitosis under indicated nutrient conditions. Box plots depict the mean (small square), median (horizontal bar), interquartile range (box) and 5-95% range (whiskers). Statistical significance was assessed using one-way ANOVA followed by Sidakholm test. Source data are provided as a Source Data file.

c Buoyant mass (black) and mass-normalized TMRE (red) traces for indicated primary cells and cell lines. Metaphase-anaphase transition (M/A) is indicated for each cell with black arrow. Note that while primary cells and several cell lines display mitochondrial hyperpolarization at the end of cell cycle, FL5.12 lymphocytes do not, indicating that mitochondrial hyperpolarization is not a general requirement for cell division.

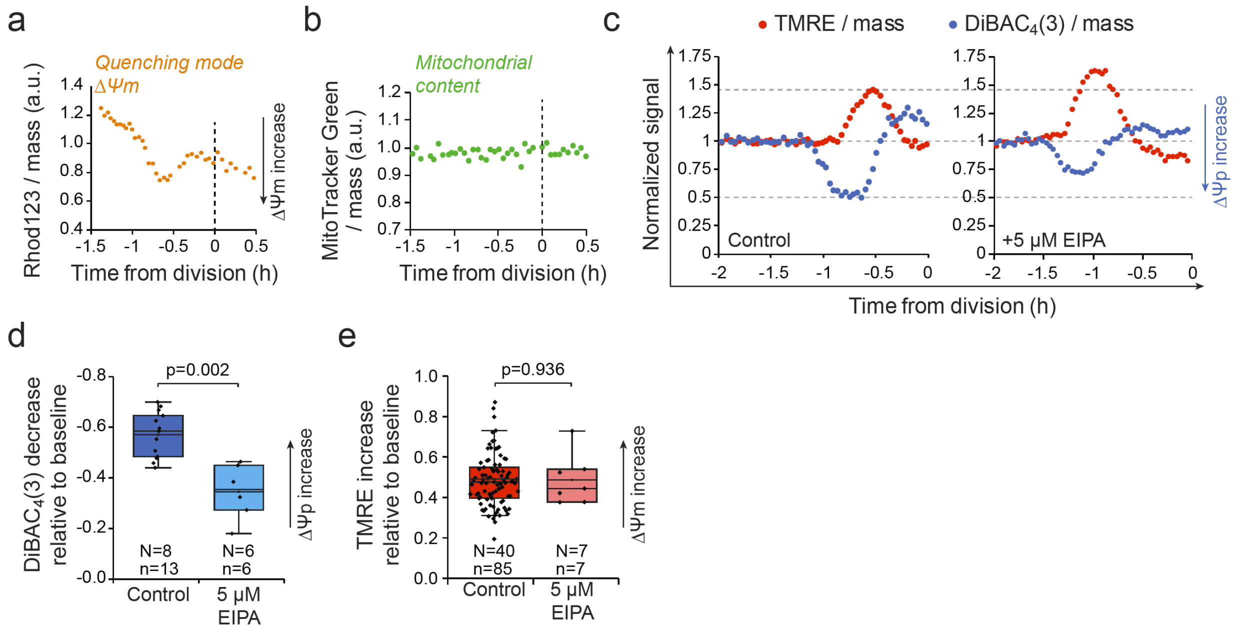

Supplementary Fig. 4 Plasma membrane hyperpolarization is coupled to mitotic cell swelling with little influence on TMRE signal.

a An additional replicate for the single-cell experiment in Fig. 1b. Mass-normalized Rhod132 trace for a L1210 cell around cell division. Cells were loaded with a quenching concentration of Rhod123 (10 µM), washed and immediately analyzed with no Rhod123 in the culture media. In the quenching mode, the fluorescence signal behavior is reversed.

b An additional replicate for the single-cell experiment in Fig. 1c. Mass-normalized MitoTracker Green (50 nM) trace for a L1210 cell around cell division.

c Mass-normalized TMRE (red, 10 nM) and DiBAC4(3) (blue, 1µM) traces for a control (left) and 5 µM EIPA treated (right) L1210 cell around cell division. Note that the DiBAC4(3) signal decrease and TMRE signal increase are not starting or ending at the same time. Also, the EIPA treatment, which inhibits mitotic cell swelling [1], reduces the DiBAC4(3) signal change but not the TMRE signal change, indicating that the plasma membrane potential changes are not significantly affecting the TMRE signal.

d, e Quantification of DiBAC4(3) **(d)** and TMRE **(e)** changes shown in panel **(c)**. Box plots depict the mean (small square), median (horizontal bar), interquartile range (IQR) (box) and 1.5 x IQR (whiskers). Statistical significances were assessed using unpaired, two-tailed Welch’s t-test. Source data are provided as a Source Data file.

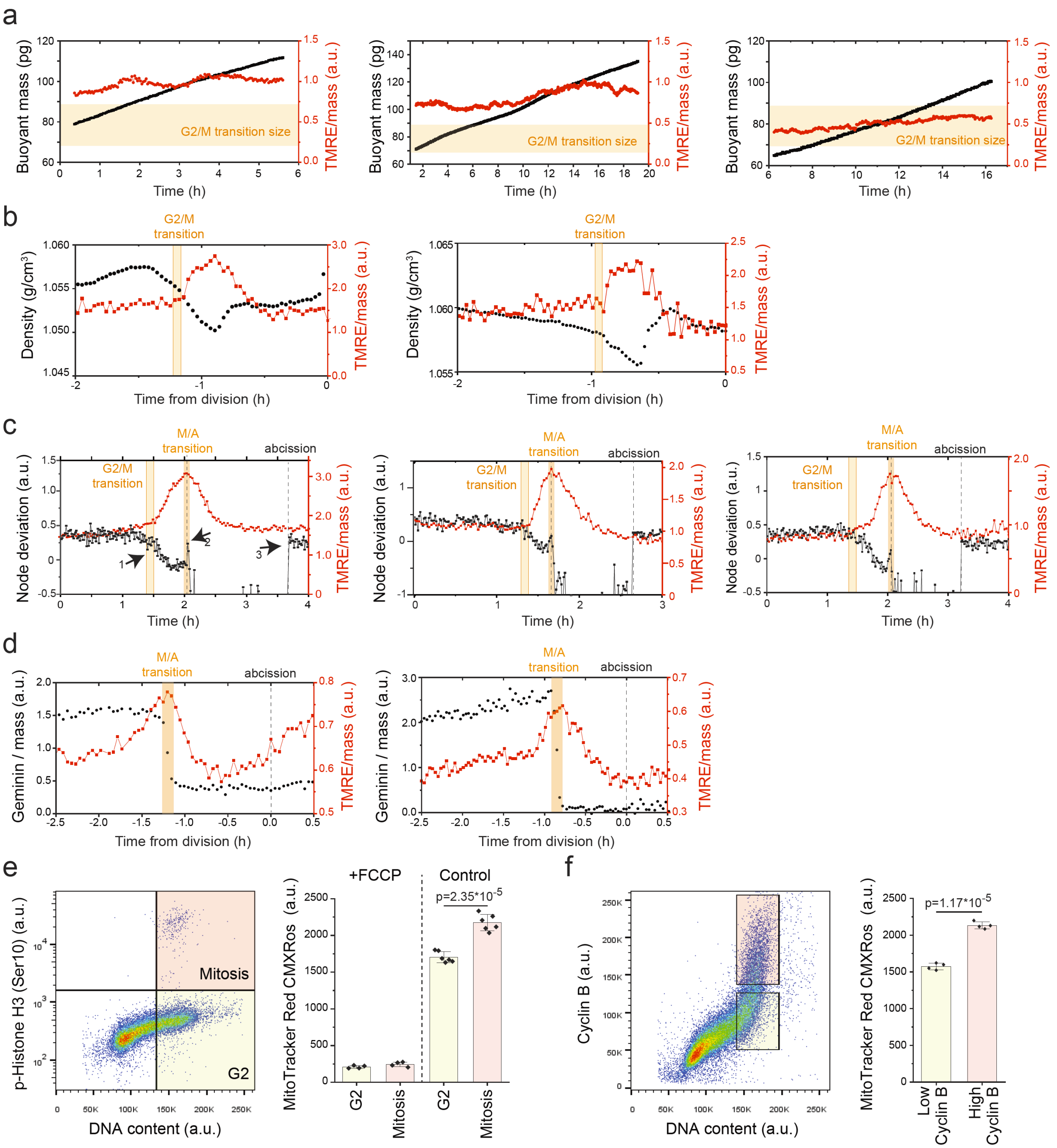

Supplementary Fig. 5 TMRE signal increases after G2/M transition until the metaphase-anaphase transition and returns back to baseline during cytokinesis.

a Additional replicates for the single-cell experiment in Fig. 1d. Buoyant mass (black) and mass-normalized TMRE (red) traces for L1210 cells treated with 2.5 µM RO-3306 to inhibit mitotic entry. Typical size for mitotic entry is illustrated with light orange area.

b Additional replicates for the single-cell experiment in Fig. 1e. Cell density (black) and mass-normalized TMRE (red) traces for L1210 cells around cell division. Mitotic entry (G2/M transition), where cell density starts to decrease[1, 13], is illustrated with light orange area.

c Node deviation (acoustic measurement dependent on cell stiffness, black) and mass-normalized TMRE (red) traces for L1210 cells around cell division. The node deviation decreases slightly after mitotic entry (1), node deviation rapidly drops as cells elongate in anaphase (2), and node deviation returns back to interphase levels at daughter cell abscission (3), thus allowing detection of G2/M transition and metaphase-anaphase transition (M/A transition)[5].

d Additional replicates single-cell for the experiment in Fig. 1g. Mass-normalized mAG-hGeminin (black) and mass-normalized TMRE (red) traces for L1210 FUCCI cells around cell division. Metaphase-anaphase transition (M/A transition) is illustrated with orange yellow area.

e *Left,* scatter plot of DNA and phosphor-Histone H3 (Ser10) staining detailing the gating used in flow cytometer to separate G2 (light yellow shading) and mitotic (light red shading) cells. *Right*, Quantifications of the MitoTracker Red CMXRos staining levels (a proxy for $\Delta\Psi m$) in G2 and mitotic cells. Mitotic control cells have significantly higher $\Delta\Psi m$. The samples on left were treated with 1 μM FCCP for 30min prior to MitoTracker staining and fixation to validate that MitoTracker Red CMXRos staining remains $\Delta\Psi m$ even after fixation. Data represents mean ± SD. Statistical significance was assessed using paired, two-tailed Student's t-test (n=6 independent cultures).

f *Left,* scatter plot of DNA and Cyclin B staining detailing the gating used in flow cytometer to separate G2&M cells with low Cyclin B (light yellow shading) and high Cyclin B (light red shading) levels. *Right*, Quantifications of the MitoTracker Red CMXRos staining levels (a proxy for $\Delta\Psi m$) in Cyclin B low and high expressing G2&M cells. Cyclin B high expressing cells have significantly higher $\Delta\Psi m$. Data represents mean ± SD. Statistical significance was assessed using paired, two-tailed Student's t-test (n=4 independent cultures). Source data are provided as a Source Data file.

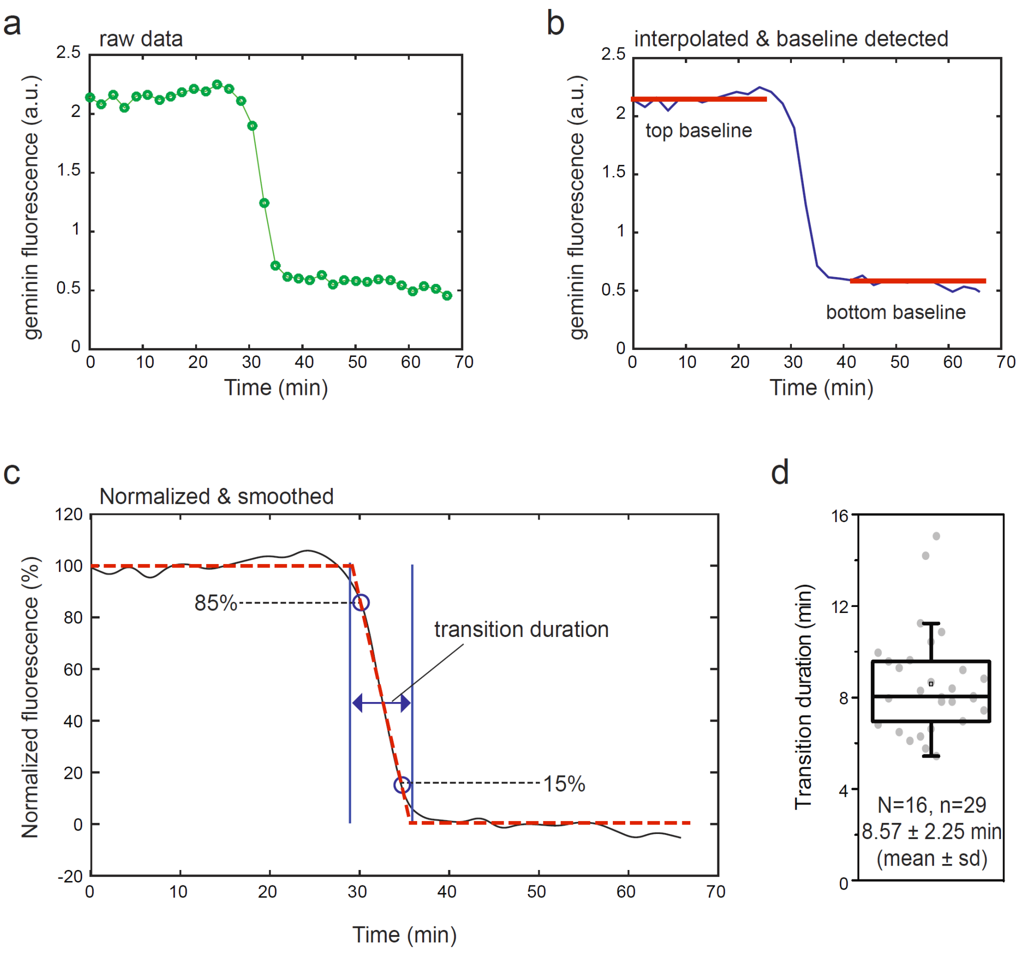

Supplementary Fig. 6 Protein degradation at metaphase-anaphase transition takes approximately 8 min in L1210 cells.

a mAG-Geminin fluorescence trace for a L1210 FUCCI cell around metaphase-anaphase transition. mAG-Geminin is degraded at metaphase-anaphase transition due to APC/C activity.

b Data in panel **(a)** after data interpolation. Averaged mAG-Geminin fluorescence level before (top baseline) and after (bottom baseline) metaphase-anaphase transition is also indicated in red.

c Data in panel **(b)** after normalization and data smoothing. This data was used to detect the transition duration by fitting a straight line between 85% and 15% mAG-Geminin fluorescence intensity points (dark blue circles) and detecting the time points where this straight line crosses the top and bottom baselines.

d Duration of protein degradation at the metaphase-anaphase transition quantified based mAG-Geminin degradation in 29 L1210 FUCCI cells. Box plots depict the mean (small square), median (horizontal bar), interquartile range (IQR) (box) and 1.5 x IQR (whiskers). Statistical significance was assessed using unpaired, two-tailed Welch's t-test.

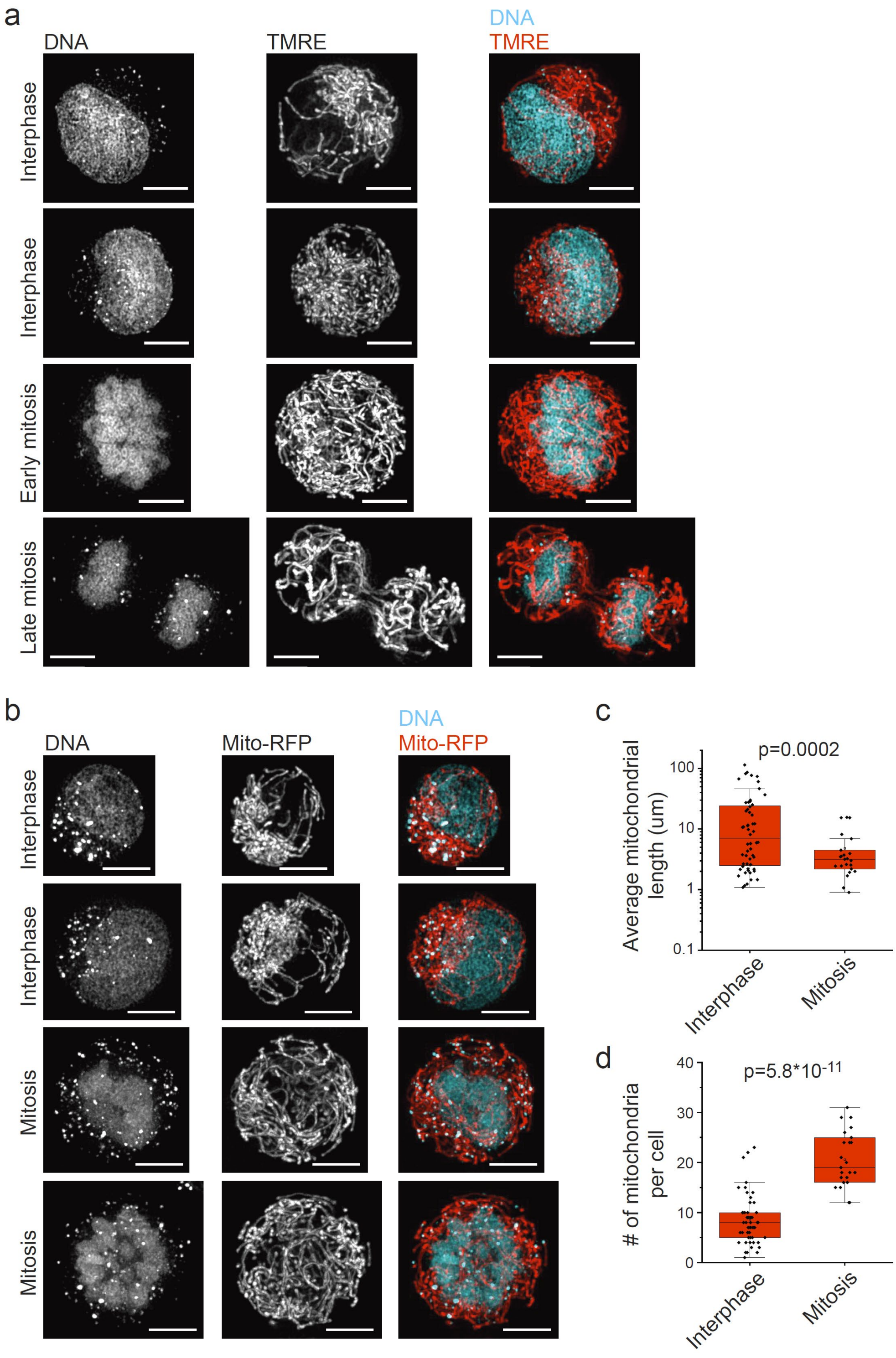

Supplementary Fig. 7 Mitochondrial morphology in L1210 cells during interphase and mitosis.

a Additional microscopy image examples for the experiment in Fig. 1h. Representative maximum intensity images of live L1210 cells stained with TMRE and PicoGreen (DNA stain). Scale bars denote 5 µm. Experiments were repeated 4 times independently with similar results.

b Representative maximum intensity images of live L1210 cells expressing mitochondria localized RFP (red) and stained with PicoGreen (DNA stain, teal). Scale bars denote 5 µm. Experiments were repeated 2 times independently with similar results.

c, d Quantifications of average length (c) and number (d) of individual mitochondria within each cell in interphase and mitosis. Mitochondrial morphology was analyzed using mitochondria localized RFP and cell cycle status as analyzed using DNA staining, as in panel **(b)**. Box plots depict the mean (small square), median (horizontal bar), interquartile range (IQR) (box) and 1.5 x IQR (whiskers). Statistical significance was assessed using unpaired, two-tailed Welch's t-test. Source data are provided as a Source Data file.

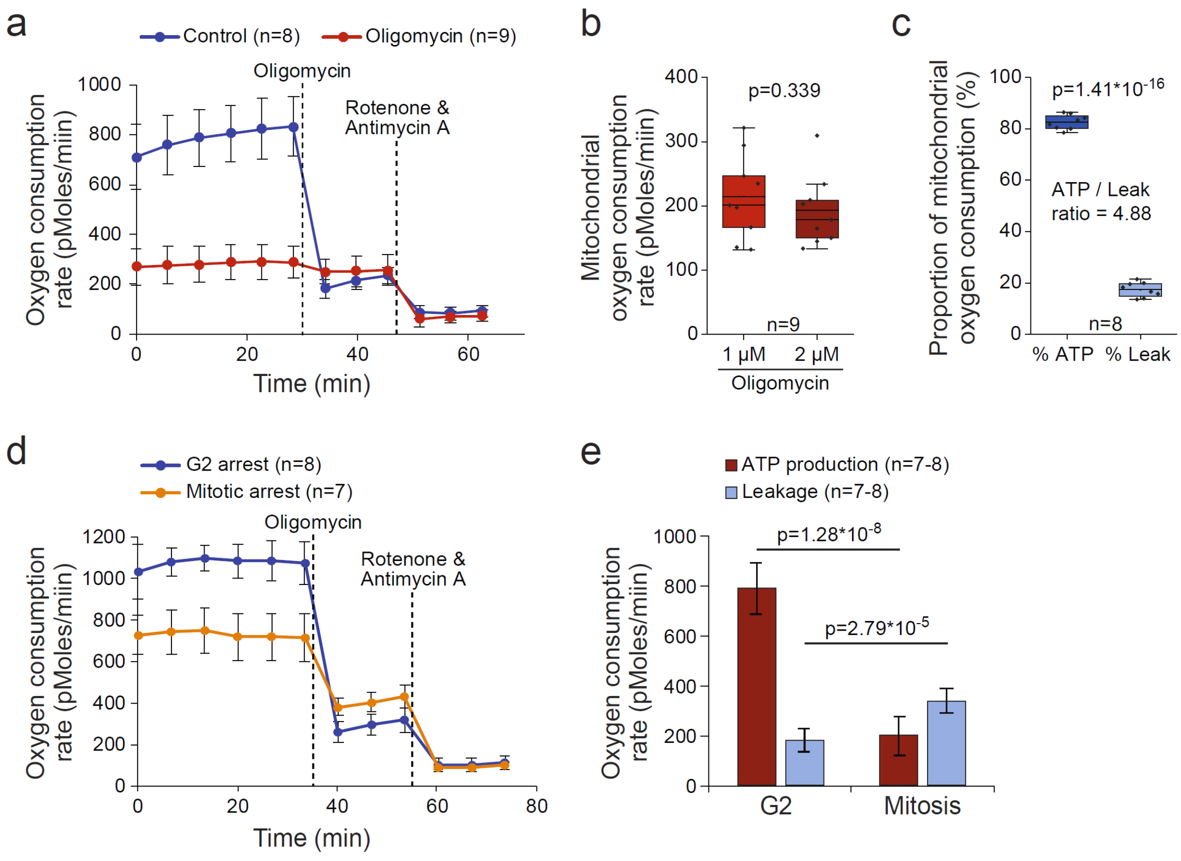

Supplementary Fig. 8 L1210 cells arrested in mitosis display lower mitochondrial ATP synthesis rates than cells arrested in G2.

a Oxygen consumption rate measurements of control L1210 cells and in L1210 cells pre-treated with 1 µM oligomycin for 4 h. Both samples were injected with 1 µM oligomycin and later with 2 µM Rotenone & 2 µM Antimycin A, as indicated by dashed lines. Data represents mean ± SD.

b Quantification of oxygen consumption rates in the 1 µM oligomycin pretreated L1210 cells before and after injection of additional oligomycin, as shown in panel **(a)**. Note that the oxygen consumption doesn't decrease further by additional oligomycin, indicating that 1 µM oligomycin is adequate to achieve maximal inhibition of ATP synthase.

c Quantification of proportional oxygen consumption rates attributable to leakage and ATP synthesis in control L1210 cells, as shown in panel **(a)**.

d Oxygen consumption rate measurements of L1210 cells arrested to G2 (blue) or to prometaphase (mitotic arrest, orange). Both samples were injected with 1 µM oligomycin and later with 2 µM Rotenone & 2 µM Antimycin A, as indicated by dashed lines. The mitotic arrest was obtained by releasing G2 arrested cells to media with STLC (kinesin motor inhibitor that arrests the cells to prometaphase) 2 h prior to measurement. Note that mitotic arrests inhibit cell growth[3] and result in mitochondrial degradation[41], which may affect the oxygen consumption rates. Data represents mean ± SD.

e Quantification of ATP synthesis and leakage rates in the G2 and mitotic arrested L1210 cells, based on the oxygen consumption measurements shown in panel **(d)**. Data represents mean ± SD (compound errors after accounting the mean values for imperfect cell cycle synchrony).
In **(a-d)**, number of independent synchronizations and cultures (n) is listed in each subpanel. Box plots in **(b, c)** depict the mean (small square), median (horizontal bar), interquartile range (IQR) (box) and 1.5 x IQR (whiskers). Source data are provided as a Source Data file. In **(b, c, e)**, statistical significances were assessed using unpaired, two-tailed Welch's t-test.

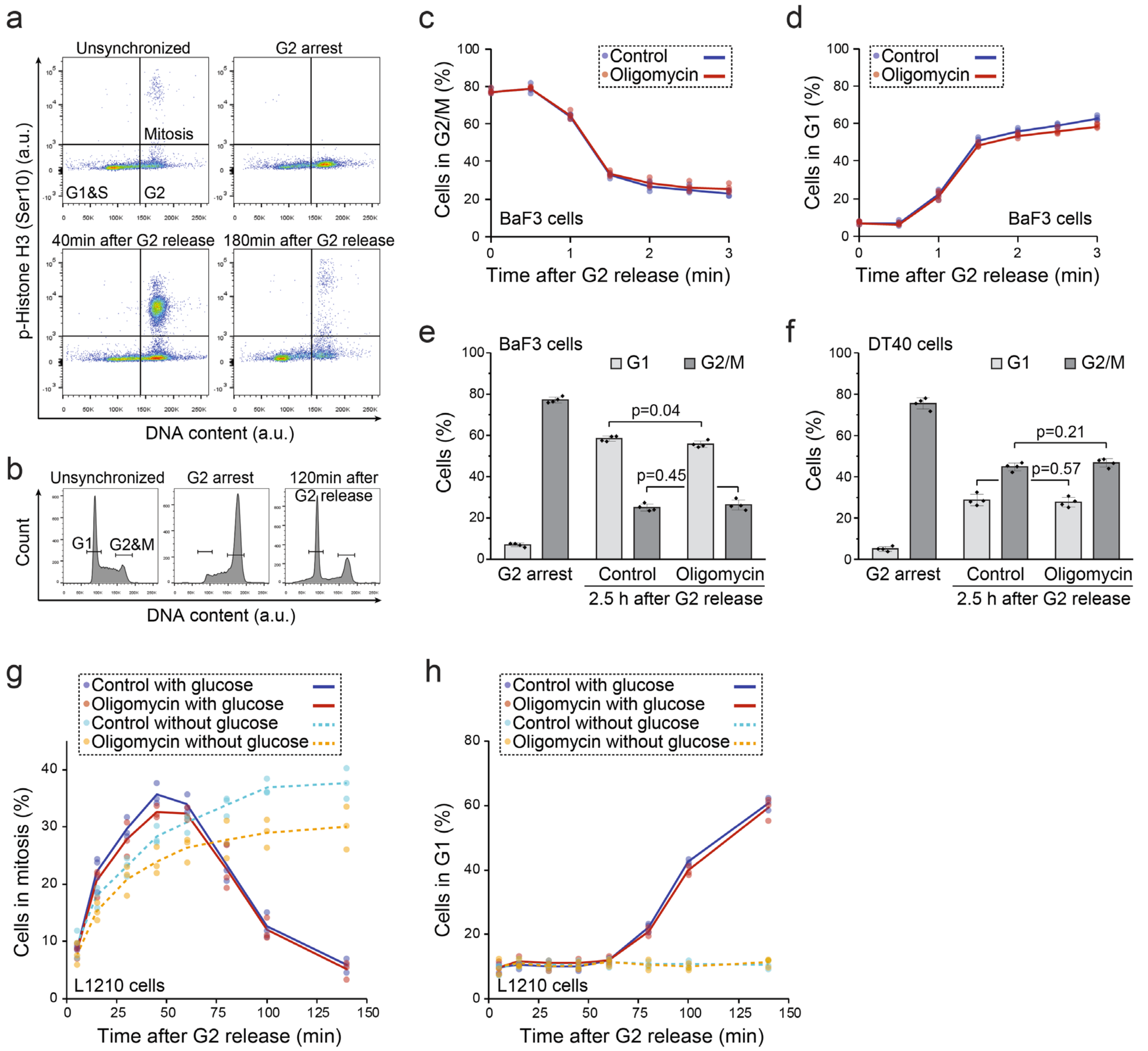

Supplementary Fig. 9 Inhibition of mitochondrial ATP synthesis has little effect on mitotic progression.

a Example flow cytometer data scatter plots of DNA and phosphor-Histone H3 (Ser10) staining together with the gating used to separate G1&S, G2 and mitotic L1210 cells. The four scatter plots represent different timepoints of cell cycle synchronization used for Fig. 3c, d and Supplementary Fig. 9g, h.

b DNA content histograms detailing the gating used in flow cytometer to separate G1 and G2&M cells. The three histograms represent different timepoints of DT40 cell cycle synchronization.

c Relative number of G2/M cells in control (blue) and 1 μM oligomycin (red) treated BaF3 cells at indicated times after release from G2 arrest. Oligomycin treatment was started 15 min before release from G2 and was maintained on cells after G2 release. Each dot represents a separate culture (n=4).

d Same as panel **(a)**, except data is indicating the relative number of G1 cells at indicated times after release from G2 arrest.
e Quantifications of BaF3 data in panels **(c, d)** for the 2.5 h timepoint after G2 release (n=4 independent cultures and synchronizations). Data represents mean ± SD. Statistical significance was assessed using unpaired, two-tailed Welch's t-test.
f Same as panel **(e)**, except experiments were carried out with DT40 chicken lymphocytes (n=4 independent cultures and synchronizations). Data represents mean ± SD. Statistical significance was assessed using unpaired, two-tailed Welch's t-test.
g Quantifications of mitotic entry in control (DMSO treated, blue) and 1 µM oligomycin-treated (red) L1210 cells in 10mM glucose containing media (solid lines), as well as the control and oligomycin treated samples in media lacking glucose (teal and yellow, respectively, dashed lines). Cells were synchronized to G2, released and collected for cell cycle analysis at indicated timepoints. Drug treatments were started and removal of glucose was done 15 min before release from G2 arrest. Each dot represents a separate culture (n=3 independent synchronizations).
h Quantifications of mitotic exit (appearance of G1 cells) for samples shown in panel **(g)**.
Source data are provided as a Source Data file.

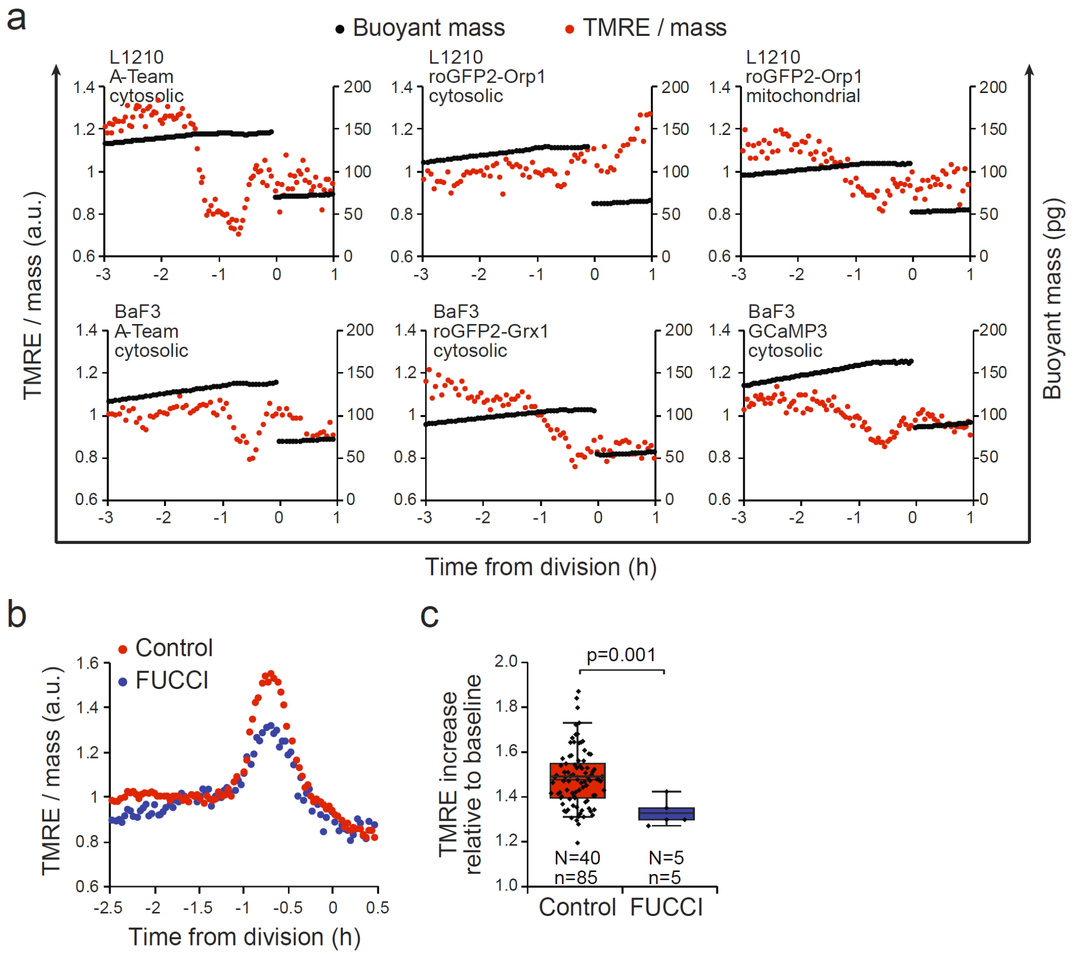

Supplementary Fig. 10 Mitotic mitochondrial hyperpolarization is perturbed by expression of exogenous proteins.

a Buoyant mass (black) and mass-normalized TMRE (red) traces for L1210 and BaF3 cells around cell division. The cells were constitutively expressing indicated genetic constructs (FRET based fluorescent sensors for various metabolites) and the TMRE hyperpolarization was no longer observed.

b Mass-normalized TMRE traces for control (wild-type, red) and FUCCI (blue) L1210 cells around cell division.

c Quantification of the mitotic TMRE increase in control (red) and FUCCI (blue) L1210 cells, as shown in panel **(b)**. Note that the FUCCI cells express mAG-Geminin under endogenous geminin promoter, and while mitochondria still hyperpolarize, this hyperpolarization is less extensive than in control (wild-type) L1210 cells. Box plots depict the mean (small square), median (horizontal bar), interquartile range (IQR) (box) and 1.5 x IQR (whiskers). Statistical significance was assessed using unpaired, two-tailed Welch's t-test. Source data are provided as a Source Data file.

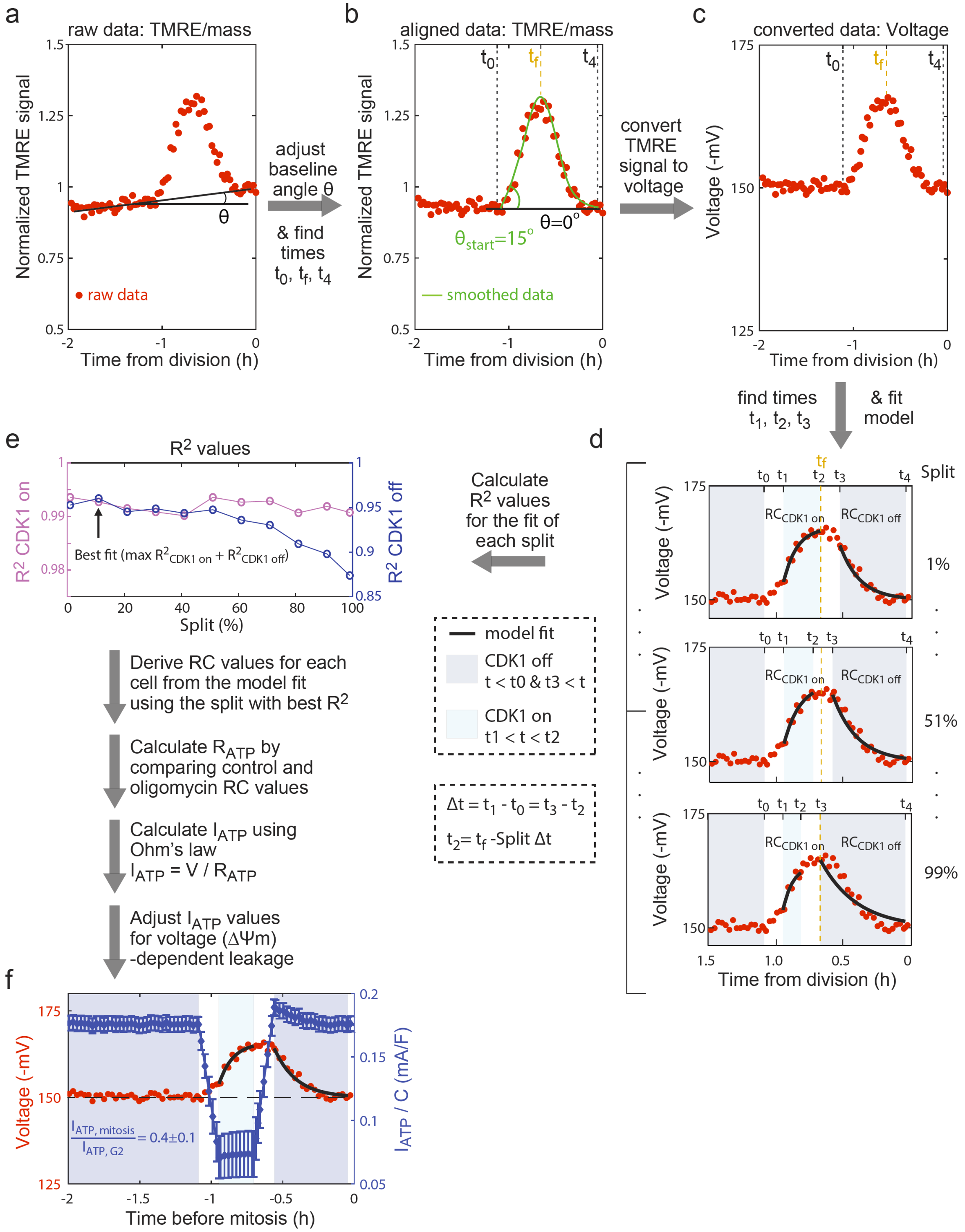

Supplementary Fig. 11 Applying electrical circuit model to mitotic TMRE data reveals the current flowing through ATP synthase.

Workflow for the modeling:

a TMRE/mass signal around mitosis is normalized to have zero baseline slope (θ). Note that the baseline slope varied between cells and slope corrections were done to both directions.

b Normalized TMRE/mass signal is fitted a smoothed curve (green) with smoothing window of 20 min. Using the smoothed curve, we find the beginning of the hyperpolarization at time t_0 where start slope is $\theta_{start} = 15^o$, the maximum of TMRE at time t_f and the end of depolarization at time t_4 where TMRE has returned to baseline (G2) levels. Cells that divided before a new baseline was clearly established were excluded from the modeling.
c TMRE/mass signal is converted to approximate voltage according to previously reported scaling between tetramethylrhodamidine ester fluorescence signal and membrane potentials[11]. $V(mV) = (TMRE/c_1)/c_2$, where $c_1 = 29.109$ and $c_2 = 0.0215$. Baseline voltage was set to -150 mV.
d Voltage signal is divided into four regions where: i) CDK1 is off (dark blue, $t_0 < t$ & $t > t_3$), ii) CDK1 transitions from off to on (white, $t_0 < t < t_1$), iii) CDK1 is on (light blue, $t_1 < t < t_2$), and iv) CDK1 transitions from off to on (white, $t_2 < t < t_3$). The transitions are set to have fixed durations ($\Delta t = t_3 - t_2 = t_1 - t_0 = 8.57\, min$), obtained by quantifying the duration of geminin protein degradation at metaphase-anaphase transition (Supplementary Fig. 6). Since t_2 (marking end of $CDK1_{on}$ region) is expected to be near the approximate metaphase-anaphase transition (t_f), yet the precise location of t_2 is unknown, eleven values $Split = 1,11,21,\dots,91,99\,\%$ were used to obtain eleven corresponding values $t_2 = t_f - Split\, \Delta t$. For each one of these values, the $CDK1_{on}$ and $CDK1_{off}$ regions were fitted with analytical solutions to the electrical circuit model (Fig. 4a) in order to obtain corresponding RC values.
e The quality of the model fitting is estimated by calculating $R^2_{CDK1\,on}$ and $R^2_{CDK1\,off}$ values for each Split. For further analysis, the RC values of each cell are derived from the Split with best fit overall for the specific cell. This procedure is repeated for each control and oligomycin treated cell to obtain population averages for $RC_{CDK1\,on}$ and $RC_{CDK1\,off}$ values, which are then used to back calculate R_{ATP} for $CDK1_{on}$ and $CDK1_{off}$ states. The R_{ATP} values are then applied to each voltage converted TMRE trace to quantify the current flowing through ATP synthase, i.e. ATP synthesis rate.
f Example of voltage (ΔΨm, red) and ATP synthesis rate (I_{ATP}/C, blue) for a single L1210 cell around cell division. Error bars depict the SEM of the R_{ATP}.
Analysis code is provided as Electrical circuit analysis code file.

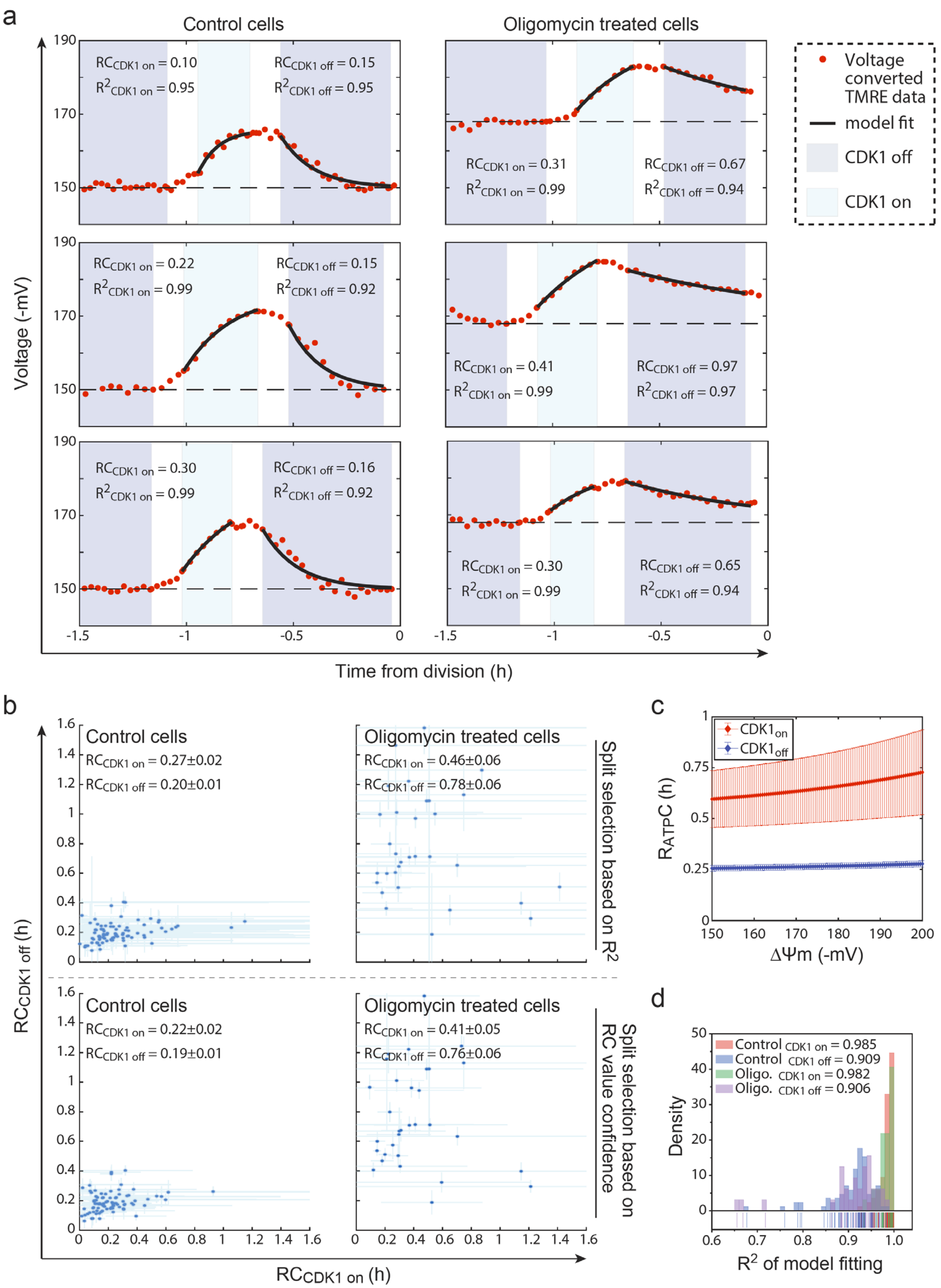

Supplementary Fig. 12 Oligomycin treatment results in increased RC values during late mitosis.

a Control (*left*) and 1 µM oligomycin (*right*) treated L1210 cell voltage traces. RC and R^2 values derived by fitting the model analytical solution (black line) to the $CDK1_{on}$ (light blue) and $CDK1_{off}$ (dark blue) regions are indicated.

b Summary of the RC values derived for each control and oligomycin treated cell when the fitting regions (Split) were selected by maximizing R^2 (*top*) or by minimizing confidence interval of the derived RC (*bottom*). Each blue dot represents a single cell and error bars depict the confidence interval of the derived RC value. The written RC values depict population mean ± SEM.
c The R_{ATP} values derived for $CDK1_{on}$ (red) and $CDK1_{off}$ (blue) states as a function of $\Delta\Psi m$. The calculated R_{ATP} values depend on $\Delta\Psi m$ due to the non-ohmic leakage scaling with $\Delta\Psi m$. Data depicts mean ± SEM (n=85 for control cells, 32 for oligomycin treated cells).
d Quality of model fitting to the TMRE data was evaluated based on fitting R^2. The histograms & rugs display R^2 values of fitting our model to the $\Delta\Psi m$ data for each control and oligomycin treated cell for both $CDK1_{on}$ and $CDK1_{off}$ states. The mean R^2 values for each group are shown in the group legend. Source data are provided as a Source Data file.

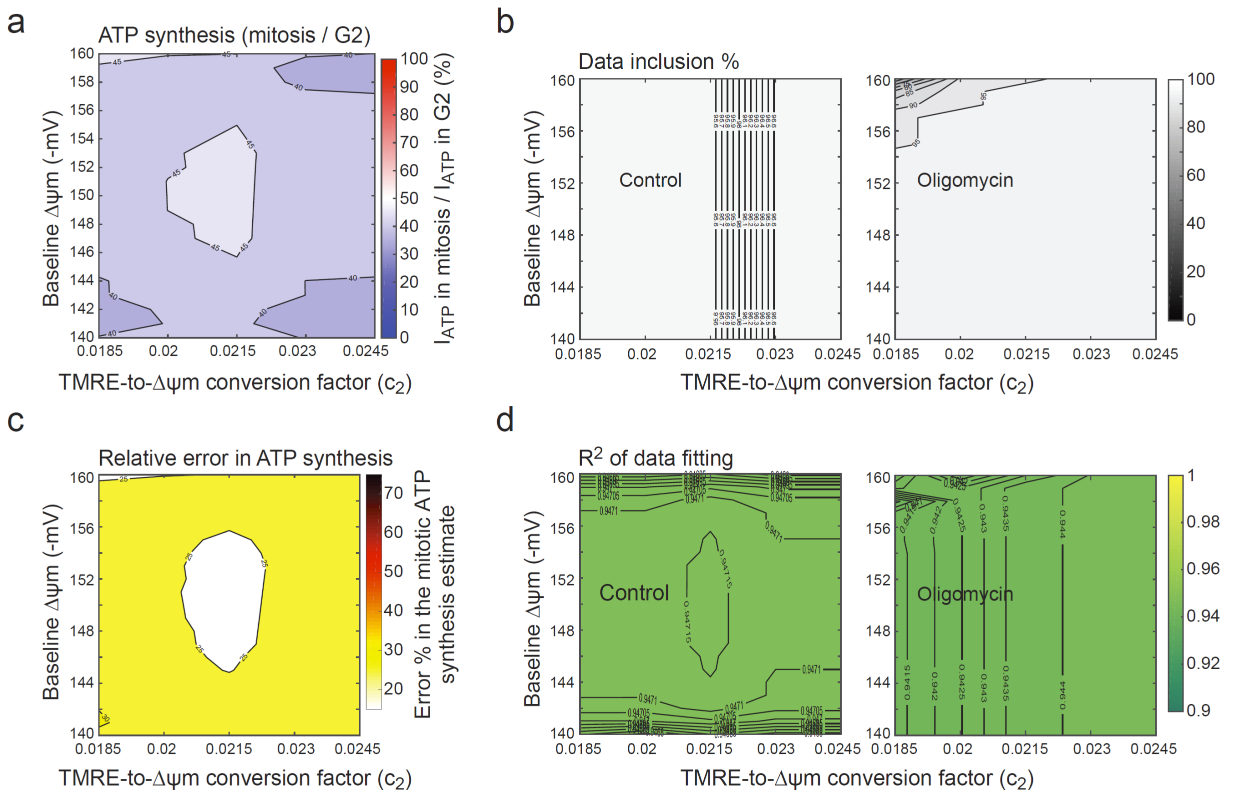

Supplementary Fig. 13 Modeling of mitotic ATP synthesis rates is not sensitive to the values used for TMRE-to-voltage conversion.

Sensitivity analysis over parameters of TMRE-to-ΔΨm conversion factor ($c_2 = 0.0185, 0.02, 0.0215, 0.0230, 0.0245$) and baseline voltage ($\Delta\Psi\text{m} = 140, 141, \ldots, 160$). The parameters for estimating the ΔΨm-dependent leakage were held fixed ($\beta = 4.88, \gamma = 0.0122$), as were the parameters used to define fitting regions, the fitting start angle and the duration of CDK1 on/off transition ($\theta_{start} = 15\ degrees, \Delta t = 8.57min$).

a Contour plot of $r_{ATP} = I_{ATP\ in\ mitosis}/I_{ATP\ in\ G2}$. Sensitivity analysis around reference values ($c_2 = 0.0215, \Delta\Psi\text{m} = 150\text{mV}$) showed $r_{ATP} = 46\%$, whereas varying c_2 or baseline voltage resulted in r_{ATP} range of $35 - 50\%$.

b Data inclusion % for control (left) and oligomycin (right) datasets.

c Relative error $\delta(I_{ATP\ in\ mitosis}/I_{ATP\ in\ G2})$ / $(I_{ATP\ in\ mitosis}/I_{ATP\ in\ G2})$. Sensitivity analysis around reference values ($c_2 = 0.0215, \Delta\Psi\text{m} = 150\text{mV}$) showed relative error $< 25\%$, whereas varying c_2 or baseline voltage resulted in errors increasing up to 30%.

d Fitting quality (R^2) for control (left) and oligomycin (right) datasets.

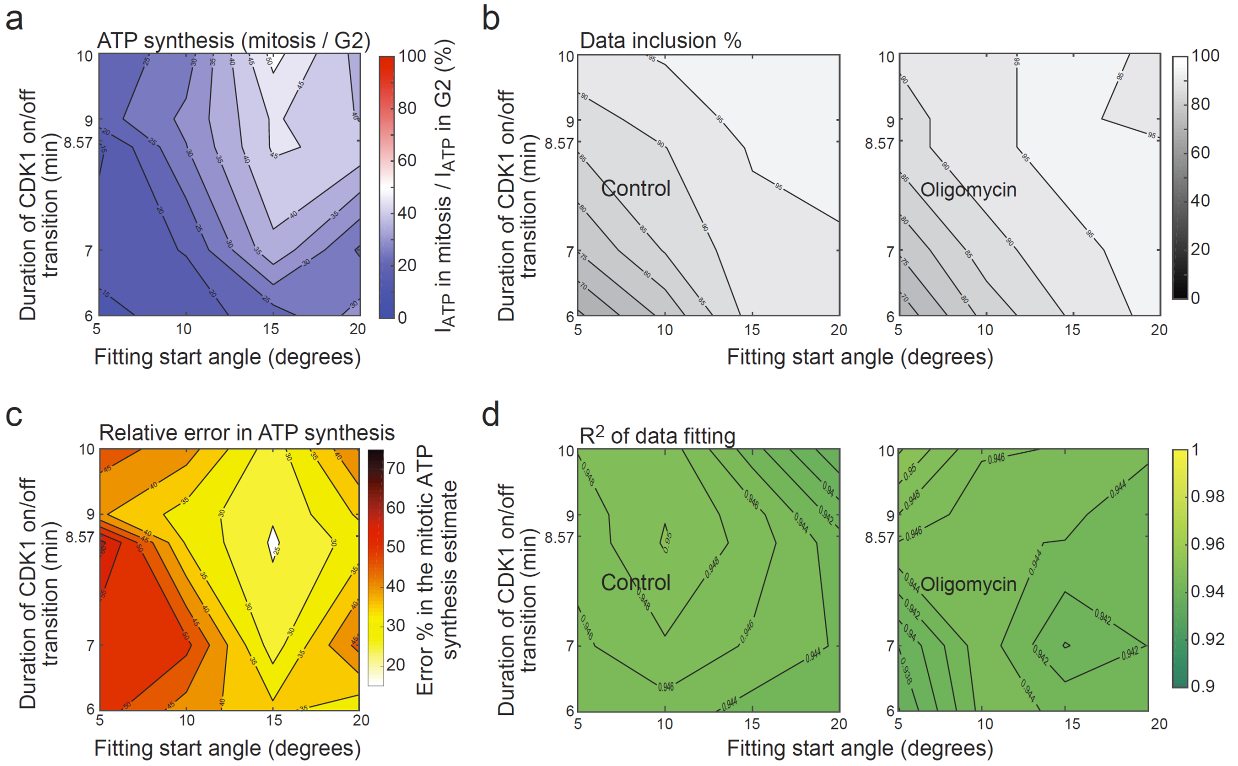

Supplementary Fig. 14 Changing fitting regions used to derive RC values results in a model prediction with more pronounced inhibition of ATP synthesis in mitosis.

Sensitivity analysis over parameters used to define fitting regions, the fitting start angle ($\theta_{start} = 5, 10, 15, 20\ degrees$) and duration of CDK1 on/off transition ($\Delta t = 6, 7, 8.57, 9, 10\ min$). The parameters for the TMRE-to-ΔΨm conversion factor and baseline voltage were held fixed ($c_2 = 0.0215, \Delta\Psi m = 150$ mV), as were the parameters for estimating the ΔΨm-dependent leakage ($\beta = 4.88, \gamma = 0.0122$).

a Contour plot of $r_{ATP} = I_{ATP\ in\ mitosis}/I_{ATP\ in\ G2}$. Sensitivity analysis around reference values ($\theta_{start} = 15\ degrees$ and $\Delta t = 8.57\ min$) showed $r_{ATP} = 46\%$, whereas varying θ_{start} or Δt resulted in r_{ATP} range of $15 - 50\%$.

b Data inclusion % for control (left) and oligomycin (right) datasets.

c Relative error $\delta(I_{ATP\ in\ mitosis}/I_{ATP\ in\ G2})$ / $(I_{ATP\ in\ mitosis}/I_{ATP\ in\ G2})$. Sensitivity analysis around reference values ($c_2 = 0.0215, \Delta\Psi m = 150$mV) showed relative error $< 25\%$, whereas varying θ_{start} or Δt resulted in errors increasing up to 55%.

d Fitting quality (R^2) for control (left) and oligomycin (right) datasets.

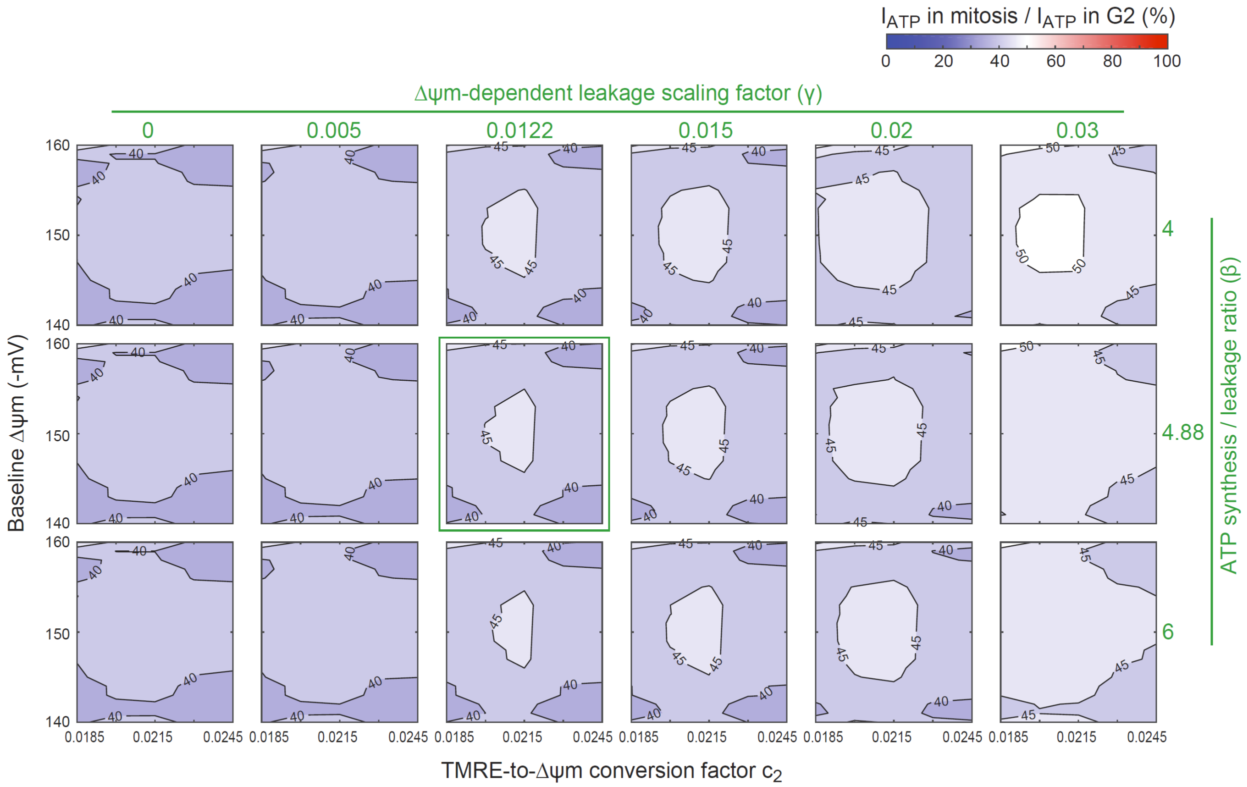

Supplementary Fig. 15 Modeling of mitotic ATP synthesis rates has little sensitivity to the values used for estimating the non-ohmic scaling of ΔΨm-dependent leakage.

Sensitivity analysis over parameters used for estimating the non-ohmic scaling of ΔΨm-dependent leakage ($\beta = 4, 4.88, 6$ *and* $\gamma = 0, 0.005, 0.0122, 0.015, 0.02, 0.03$). The parameters used to define fitting regions, the fitting start angle and the duration of CDK1 on/off transition ($\theta_{start} = 15\ degrees, \Delta t = 8.57 min$), were held fixed. The parameters for the TMRE-to-ΔΨm conversion were varied in similar range as in Supplementary Fig. 13. Data depicts contour plots of $r_{ATP} = I_{ATP\ in\ mitosis}/I_{ATP\ in\ G2}$. The green box indicates the reference values ($\beta = 4.88, \gamma = 0.0122$). Overall, varying parameters used for estimating the non-ohmic scaling of ΔΨm-dependent leakage resulted in r_{ATP} range of $35 - 55\%$.

Supplementary References

1. Son, S. *et al.* Resonant microchannel volume and mass measurements show that suspended cells swell during mitosis. *J Cell Biol* **211**, 757-763 (2015).
2. Son, S. *et al.* Direct observation of mammalian cell growth and size regulation. *Nat Methods* **9**, 910-912 (2012).
3. Miettinen, T.P., Kang, J.H., Yang, L.F. & Manalis, S.R. Mammalian cell growth dynamics in mitosis. *Elife* **8** (2019).
4. Burg, T.P. *et al.* Weighing of biomolecules, single cells and single nanoparticles in fluid. *Nature* **446**, 1066-1069 (2007).
5. Kang, J.H. *et al.* Noninvasive monitoring of single-cell mechanics by acoustic scattering. *Nat Methods* **16**, 263-269 (2019).
6. Perry, S.W., Norman, J.P., Barbieri, J., Brown, E.B. & Gelbard, H.A. Mitochondrial membrane potential probes and the proton gradient: a practical usage guide. *Biotechniques* **50**, 98-115 (2011).
7. Farkas, D.L., Wei, M.D., Febbroriello, P., Carson, J.H. & Loew, L.M. Simultaneous imaging of cell and mitochondrial membrane potentials. *Biophys J* **56**, 1053-1069 (1989).
8. Ward, M.W., Rego, A.C., Frenguelli, B.G. & Nicholls, D.G. Mitochondrial membrane potential and glutamate excitotoxicity in cultured cerebellar granule cells. *J Neurosci* **20**, 7208-7219 (2000).
9. Nicholls, D.G. & Ward, M.W. Mitochondrial membrane potential and neuronal glutamate excitotoxicity: mortality and millivolts. *Trends Neurosci* **23**, 166-174 (2000).
10. Ehrenberg, B., Montana, V., Wei, M.D., Wuskell, J.P. & Loew, L.M. Membrane-Potential Can Be Determined in Individual Cells from the Nernstian Distribution of Cationic Dyes. *Biophysical Journal* **53**, 785-794 (1988).
11. Gerencser, A.A. *et al.* Quantitative measurement of mitochondrial membrane potential in cultured cells: calcium-induced de- and hyperpolarization of neuronal mitochondria. *J Physiol* **590**, 2845-2871 (2012).
12. Zorova, L.D. *et al.* Functional Significance of the Mitochondrial Membrane Potential. *Biochem Mosc Suppl S* **12**, 20-26 (2018).
13. Zlotek-Zlotkiewicz, E., Monnier, S., Cappello, G., Le Berre, M. & Piel, M. Optical volume and mass measurements show that mammalian cells swell during mitosis. *J Cell Biol* **211**, 765-774 (2015).
14. Miettinen, T.P. *et al.* Identification of transcriptional and metabolic programs related to mammalian cell size. *Curr Biol* **24**, 598-608 (2014).
15. Miettinen, Teemu P. & Björklund, M. Cellular Allometry of Mitochondrial Functionality Establishes the Optimal Cell Size. *Developmental Cell* **39** (2016).
16. Kitami, T. *et al.* A Chemical Screen Probing the Relationship between Mitochondrial Content and Cell Size. *Plos One* **7** (2012).

17. Gan, Z., Audi, S.H., Bongard, R.D., Gauthier, K.M. & Merker, M.P. Quantifying mitochondrial and plasma membrane potentials in intact pulmonary arterial endothelial cells based on extracellular disposition of rhodamine dyes. *Am J Physiol-Lung C* **300**, L762-L772 (2011).
18. Nicholls, D.G. & Budd, S.L. Mitochondria and neuronal survival. *Physiol Rev* **80**, 315-360 (2000).
19. Gavet, O. & Pines, J. Progressive activation of CyclinB1-Cdk1 coordinates entry to mitosis. *Dev Cell* **18**, 533-543 (2010).
20. Lindqvist, A., Rodriguez-Bravo, V. & Medema, R.H. The decision to enter mitosis: feedback and redundancy in the mitotic entry network. *J Cell Biol* **185**, 193-202 (2009).
21. Hegarat, N., Rata, S. & Hochegger, H. Bistability of mitotic entry and exit switches during open mitosis in mammalian cells. *Bioessays* **38**, 627-643 (2016).
22. Brand, M.D. & Nicholls, D.G. Assessing mitochondrial dysfunction in cells. *Biochem J* **435**, 297-312 (2011).
23. Nicholls, D.G. Mitochondrial membrane potential and aging. *Aging Cell* **3**, 35-40 (2004).
24. Neufer, P.D. The Bioenergetics of Exercise. *Cold Spring Harb Perspect Med* **8** (2018).
25. Padmaraj, D., Pande, R., Miller, J.H., Jr., Wosik, J. & Zagozdzon-Wosik, W. Mitochondrial membrane studies using impedance spectroscopy with parallel pH monitoring. *PLoS One* **9**, e101793 (2014).
26. Lemeshko, V.V. VDAC electronics: 3. VDAC-Creatine kinase-dependent generation of the outer membrane potential in respiring mitochondria. *Biochim Biophys Acta* **1858**, 1411-1418 (2016).
27. Diogo, C.V., Grattagliano, I., Oliveira, P.J., Bonfrate, L. & Portincasa, P. Re-wiring the circuit: mitochondria as a pharmacological target in liver disease. *Curr Med Chem* **18**, 5448-5465 (2011).
28. Dimroth, P., Kaim, G. & Matthey, U. Crucial role of the membrane potential for ATP synthesis by F1Fo ATP synthases. *J Exp Biol* **203**, 51-59 (2000).
29. Mitchell, P. & Moyle, J. Estimation of membrane potential and pH difference across the cristae membrane of rat liver mitochondria. *Eur J Biochem* **7**, 471-484 (1969).
30. Robb, E.L. *et al.* Control of mitochondrial superoxide production by reverse electron transport at complex I. *J Biol Chem* **293**, 9869-9879 (2018).
31. Scialo, F., Fernandez-Ayala, D.J. & Sanz, A. Role of Mitochondrial Reverse Electron Transport in ROS Signaling: Potential Roles in Health and Disease. *Front Physiol* **8**, 428 (2017).
32. Nobes, C.D., Brown, G.C., Olive, P.N. & Brand, M.D. Non-ohmic proton conductance of the mitochondrial inner membrane in hepatocytes. *J Biol Chem* **265**, 12903-12909 (1990).
33. Jastroch, M., Divakaruni, A.S., Mookerjee, S., Treberg, J.R. & Brand, M.D. Mitochondrial proton and electron leaks. *Essays Biochem* **47**, 53-67 (2010).

34. Brand, M.D. Mitochondrial generation of superoxide and hydrogen peroxide as the source of mitochondrial redox signaling. *Free Radical Bio Med* **100**, 14-31 (2016).
35. Loew, L.M., Tuft, R.A., Carrington, W. & Fay, F.S. Imaging in five dimensions: time-dependent membrane potentials in individual mitochondria. *Biophys J* **65**, 2396-2407 (1993).
36. Conn, A.R., Gould, N.I.M. & Toint, P.L. *Trust-region methods*. (Society for Industrial and Applied Mathematics, Philadelphia, PA; 2000).
37. Nicholls, D.G. The influence of respiration and ATP hydrolysis on the proton-electrochemical gradient across the inner membrane of rat-liver mitochondria as determined by ion distribution. *Eur J Biochem* **50**, 305-315 (1974).
38. Nicholls, D.G. The effective proton conductance of the inner membrane of mitochondria from brown adipose tissue. Dependency on proton electrochemical potential gradient. *Eur J Biochem* **77**, 349-356 (1977).
39. Nicholls, D.G. Hamster brown-adipose-tissue mitochondria. The control of respiration and the proton electrochemical potential gradient by possible physiological effectors of the proton conductance of the inner membrane. *Eur J Biochem* **49**, 573-583 (1974).
40. Wang, Z. *et al.* Cyclin B1/Cdk1 coordinates mitochondrial respiration for cell-cycle G2/M progression. *Dev Cell* **29**, 217-232 (2014).
41. Domenech, E. *et al.* AMPK and PFKFB3 mediate glycolysis and survival in response to mitophagy during mitotic arrest. *Nat Cell Biol* **17**, 1304-1316 (2015).
42. Harbauer, A.B. *et al.* Mitochondria. Cell cycle-dependent regulation of mitochondrial preprotein translocase. *Science* **346**, 1109-1113 (2014).
43. Picard, M., McEwen, B.S., Epel, E.S. & Sandi, C. An energetic view of stress: Focus on mitochondria. *Front Neuroendocrinol* **49**, 72-85 (2018).
44. Chretien, D. *et al.* Mitochondria are physiologically maintained at close to 50 degrees C. *PLoS Biol* **16**, e2003992 (2018).
45. Rodenfels, J., Neugebauer, K.M. & Howard, J. Heat Oscillations Driven by the Embryonic Cell Cycle Reveal the Energetic Costs of Signaling. *Dev Cell* **48**, 646-658 e646 (2019).
