# Supplementary material for: Monitoring and modeling of lymphocytic leukemia cell bioenergetics reveals decreased ATP synthesis during cell division
Source: Nat Commun. 2020 Oct 5;11:4983. doi: 10.1038/s41467-020-18769-y (PMC7536222; doi:10.1038/s41467-020-18769-y)
Supplement: Supplementary file 3 — Electrical circuit analysis code [file 41467_2020_18769_MOESM3_ESM.zip › RC_fitting_code_to_data_v2/Instructions to run codes for analysis.docx]

**Instructions for running the codes for analysis**

**Prepared by**: Georgios ‘Yorgos’ Katsikis

**Code Run on**: MacBook Pro 2015, 2.2 GHz Quad-Core Intel Core i7 16 GB 1600 MHz DDR3 on Operating system macOS Catalina.

**Software used**: MATLAB 2019b (Version 9.7) with Curve Fitting Toolbox Version 3.5.10

The code was also tested on Windows-based PC running MATLAB 2017b.

**Data included**: The raw data files contain all original experimental TMRE data used in the electrical circuit model.

**Folder Structure**: Opening the zipped file gives the following folder structure:

| Initial structure (before running code RunAll.m) |
| --- |
| 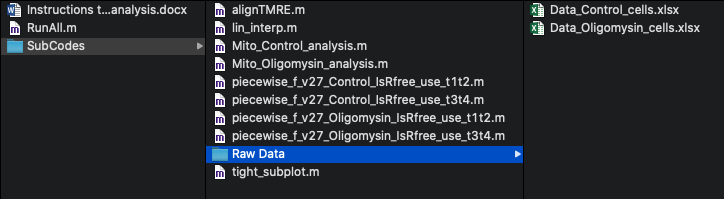 |
| Final structure (after running code Runall.m) |
| 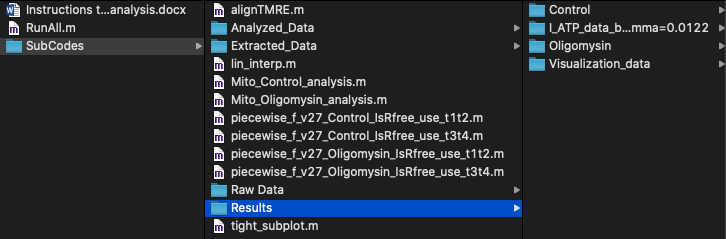 |

**Brief description of folders and files**

- The raw data files from experimental measurements of Control and Oligomysin cells are respectively:

*SubCodes/Raw Data/Data_Control_cells.xlsx*

*SubCodes/Raw Data/Data_Oligomysin_cells.xlsx*

Each .xlsx file contains a tab with a dataset for a single cell undergoing mitosis. Each dataset has four columns 1) mass(picograms), 2)time (h), 3) TMRE/mass (a.u.), 4) Time from division (h).

- The code *RunAll.m* executes the three main Matlab code analysis files:

*SubCodes/Mito_Control_analysis.m*

*SubCodes/Oligomysin/Mito_Oligomysin_analysis.m*

*SubCodes/alignTMRE_tf.m*.

Inside the analysis codes the 3^rd^ column of the raw data files (TMRE/mass) is normalized so that its average value is unit “1”.

- The folder *SubCodes/Results* contains the outputs. In particular, it contains four pieces of informations divided in four corresponding subfolders:

i,ii) the fittings in the form of numerical data and images from Control, and Oligomysin data respectively in

*SubCodes/Results/Control*

*SubCodes/Results/Oligomysin*

iii) the data of traces of ATP currents in *SubCodes/Results/Data_for_alignment_Vo150_c20.0215*

(the numbers in the name correspond to the specific parameter values)

iv) visualization data showing images of fittings and calculated ATP currents as well as data of RC values in

*SubCodes/Results/* *Visualization_data/Visualization_Vo150_c20.0215*

(the numbers in the name correspond to the specific parameter values)

- The files *SubCodes/tight_subplot.m* and *SubCodes/lin_interp.m* perform plotting and interpolation function respectively and are not meant to be changed by the user. The folder *Analyzed Data* contains temporary results that get generated during running the codes and are also not meant to be changed by the user.

**List of inputs for codes**

Below we write the list of inputs of codes. These input are key variables are not defined globally in *RunAll.m* so they have to be changed appropriately in both codes:

*SubCodes/Control/Mito_Control_analysis.m*

*SubCodes/Oligomysin/Mito_Oligomysin_analysis.m*.

The inputs highlighted in bold are the most important for modelling purposes and are derived from experiments and literature.

| **Inputs** | | | |
| --- | --- | --- | --- |
| Description | Variable | Description | Variable |
| Vo_forced | **Voltage Baseline for Controls (mV)** | dur_time | Half-duration for searching transition regions of CKD1 activity (exact value does not matter) |
| c2_exp_forced | **c_2_ exponent for TMRE-Voltage conversion** | smooth_factor | smooth factor for linear and backward slopes |
| minutes_smooth | smoothing filter in minutes for curves | split_array | Array of distinct split values, for picking the potential regions where the transition of CDK1 activity take place |
| N_interpolation_points | number of interpolation points for curves after smoothing | do_slope_correction | **Variable determining (if 1) if manual slope correction for each dataset will be done** |
| N_fall | Number of search points for CDK1-off region | angle_start_look | **angle (deg) to identify the onset of region CDK1-on** |
| N_rise | Number of search points for CDK1-on region | angle_end_look | **angle (deg) to identify the end of the curve after onset of region CDK1-off** |
| N_dev | Offset of search points for CDK1-on region | duration_search_region | Duration search region |
| Deltat_backward | Duration of baseline before start of CDK1-on phase | Deltat_forward | Duration of baseline after end of CDK1-off phase |
| lin_increase | **Duration of transition from CDK1 off to on** | lin_decrease | **Duration of transition from CDK1 on to off** |
| beta | **Ratio between ATP synthesis and leakage** | gamma | **Membrane Potential dependent leakage scaling factor** |

**Steps for running the codes**

To perform the analysis, the user needs to run the code *RunAll.m*. No other action is needed by the user as the *RunAll.m* executes four steps where it runs the three subcodes:

*SubCodes/Mito_Control_analysis.m*

*SubCodes/Oligomysin/Mito_Oligomysin_analysis.m*

*SubCodes/alignTMRE_tf.m*.

Note that *RunAll.m* has some variables (*in lines:32-26, 56-58, 75-79*) related to specific functionaties of the three subcodes. The user does not necessarily need to change this. If prompted by MATLAB to “change folder” or “add path”, please select “change folder” (see below).


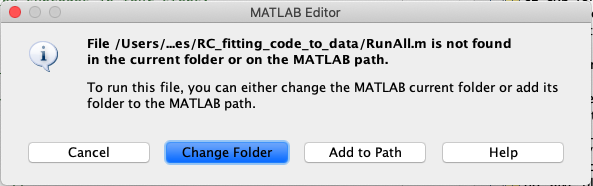


**What code *RunAll.m* does in four steps**:

- In step 1, *RunAll.m* runs *SubCodes/Mito_Control_analysis.m* which processes the Control data. In particular, the subcode:

i) extracts raw control data from Excel format and saves them in Matlab format in *SubCodes/Extracted_Data/Control* making a series of *File_1.mat* to .. *File_124.mat*. This is done through variable do_extract_Control_data=1 (in *line:32 of RunAll.m*)

ii) does the RC fits and saves data to *SubCodes/Results/Control/Datac20.0215VC150/* where it creates subfolders */Dataset1*, */Dataset2* etc. In each one of these subfolders, the code creates data file *Data Control #1.mat* and image file *Data Control #1.png* and *Data Control #1.pdf* and so on for every dataset.

This is done through variable do_analyze_Control_data=1; (in *line:33 of RunAll.m*) and the saving of the plot is optional through do_save_plots=1; (in *line:36 of RunAll.m*)

- In step 2, *RunAll.m* runs *SubCodes/Mito_Oligomysin_analysis.m* which processes the Oligomysin data doing similar actions as in step 2, saving extracted data in *SubCodes/Extracted_Data/Oligomysin* and RC fits in *SubCodes/Results/Oligomysin/Datac20.0215VC150/.* Note that for the analysis of oligomysin datasets, the baseline is set by adding to the baseline of Control (V=150mV) an increment derived from experiments and literature log(1.472)/c_2_ (supplementary note). This is done through variables do_extract_Oligomysin_data=1 , do_analyze_Oligomysin_data=1; (in *lines:56, 57 respectively of RunAll.m*).
- In step 3, *RunAll.m* runs *SubCodes/Mito_Control_analysis.m* again to compile the analyzed data from steps 1 and 2. In particular, the subcode:

i) calculates all the statistics for the RC values and makes relevant plots that it saves at *SubCodes/Results/Visualization_data/Visualization_Vo150_c20.0215/*.

ii) it saves the RC values in a single file as:

*SubCodes/Results/Visualization_data/Visualization_Vo150_c20.0215/RC_values.mat*

iii) it uses the RC values to calculate the resistance R_A_C for ATP, and calculate the currents I_ATP_ and all the associated statistical errors, all of which are plotted and saved at *SubCodes /Results/Visualization_data/Visualization_Vo150_c20.0215/*

iv) it saves data for calculated currents I_ATP_ for each individual Control dataset in SubCodes /Results/I_ATP_data _ beta=4.88_gamma=0.0122/Data_for_alignment_Vo150_c20.0215/ as *File_1.mat* etc.

- In step 4, *RunAll.m* runs *SubCodes/alignTMRE_tf.m* which takes the individual data for I_ATP_ currents and aligns them along time so that we obtain the averaged I_ATP_ across all control over time and makes the plots.

**Run time:** approximately 30 minutes in total.

**Notes:** Run time can be significantly reduced if user deactivates saving plots, by setting the following variable do_save_plots from 1 to 0 (*in line:79*)

If do_save_plots=0, the code just produces numerical data without saving plots.

**Outputs for codes**

We present representative outputs that the codes give in the order they are generated as the user progresses from Step 1 to Step 4.

In Step 1, the code gives data and images from the analysis of every individual dataset of Control cells. In the image below, we show the fits on all eleven different split values. The panel on the bottom right shows the R^2^ values for the CDK1-on regime in light pink color (labelled here as “Spike”) and CKD1-off regime in light blue color (labelled here as “Fall-off”). The code selects the split that yield the best mean R^2^ value from CDK1-on and CDK1-off regimes (here the split on the top right corner). Note that the splits that yield IsR value above a threshold of around 200mV are rejected.


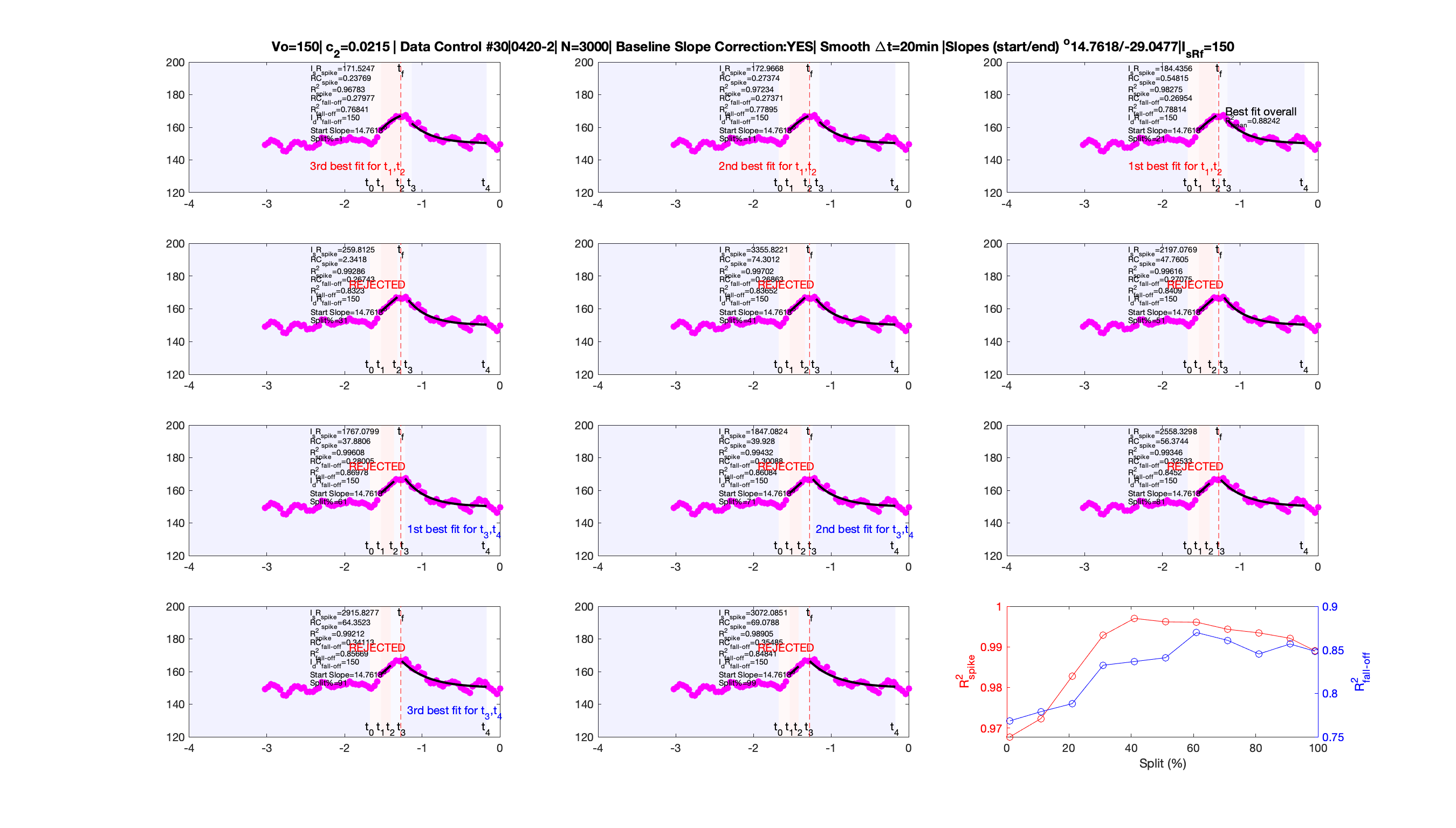
 In Step 2, the code gives data and images from the analysis of every individual dataset of Oligomysin cells (example below) in exactly the same way as in step 1 for Control cells.


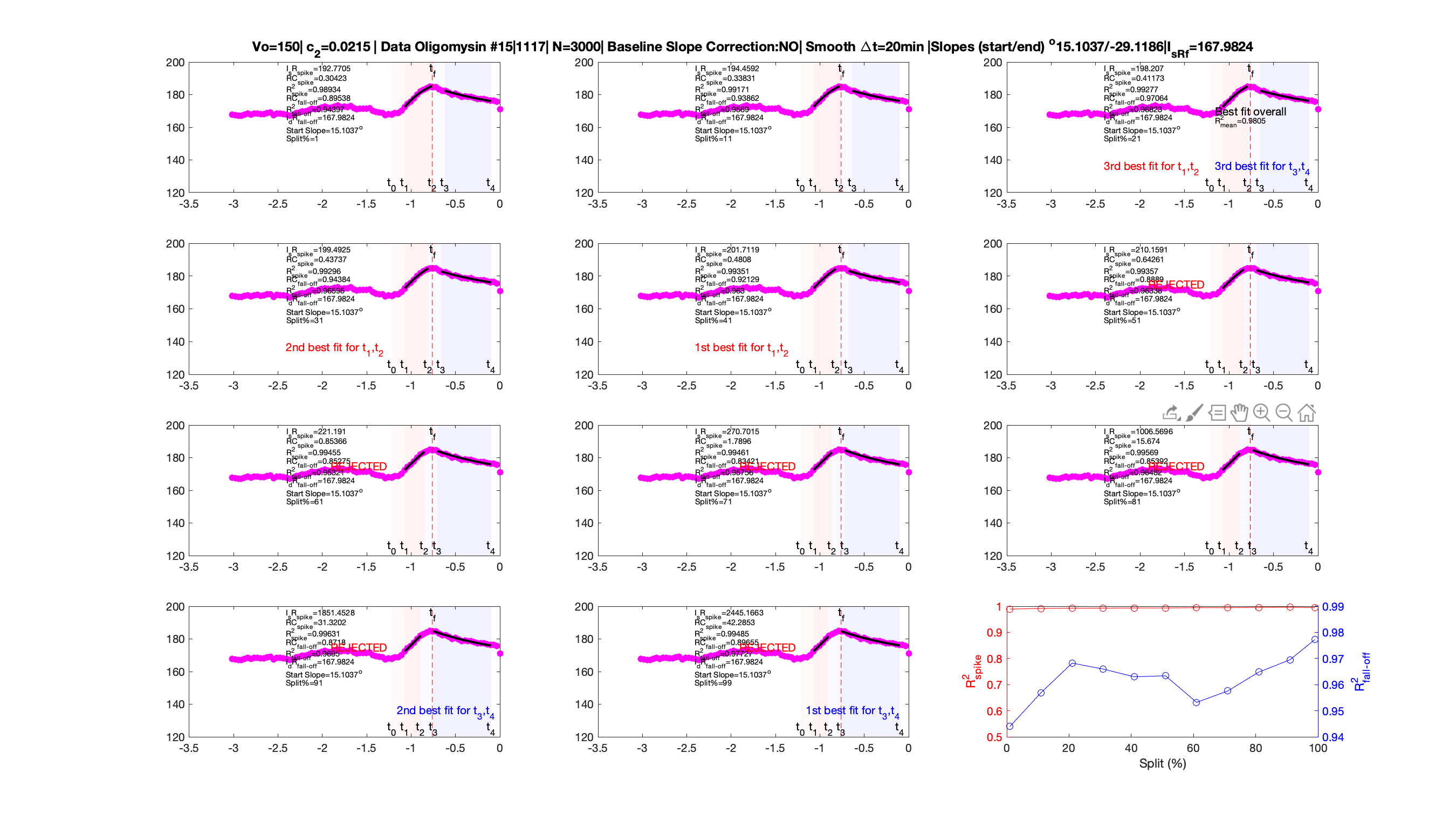


In step 3, the code provides statistics for RC values of selected splits of Control and Oligomysin cells both in file *SubCodes/Results/Visualization_data/Visualization_Vo150_c20.0215/RC_values.mat* and in the image file as shown below. When user opens file */RC_values.mat*, *RC_s* refers to CDK1-on state and *RC_f* to CDK1-off state.


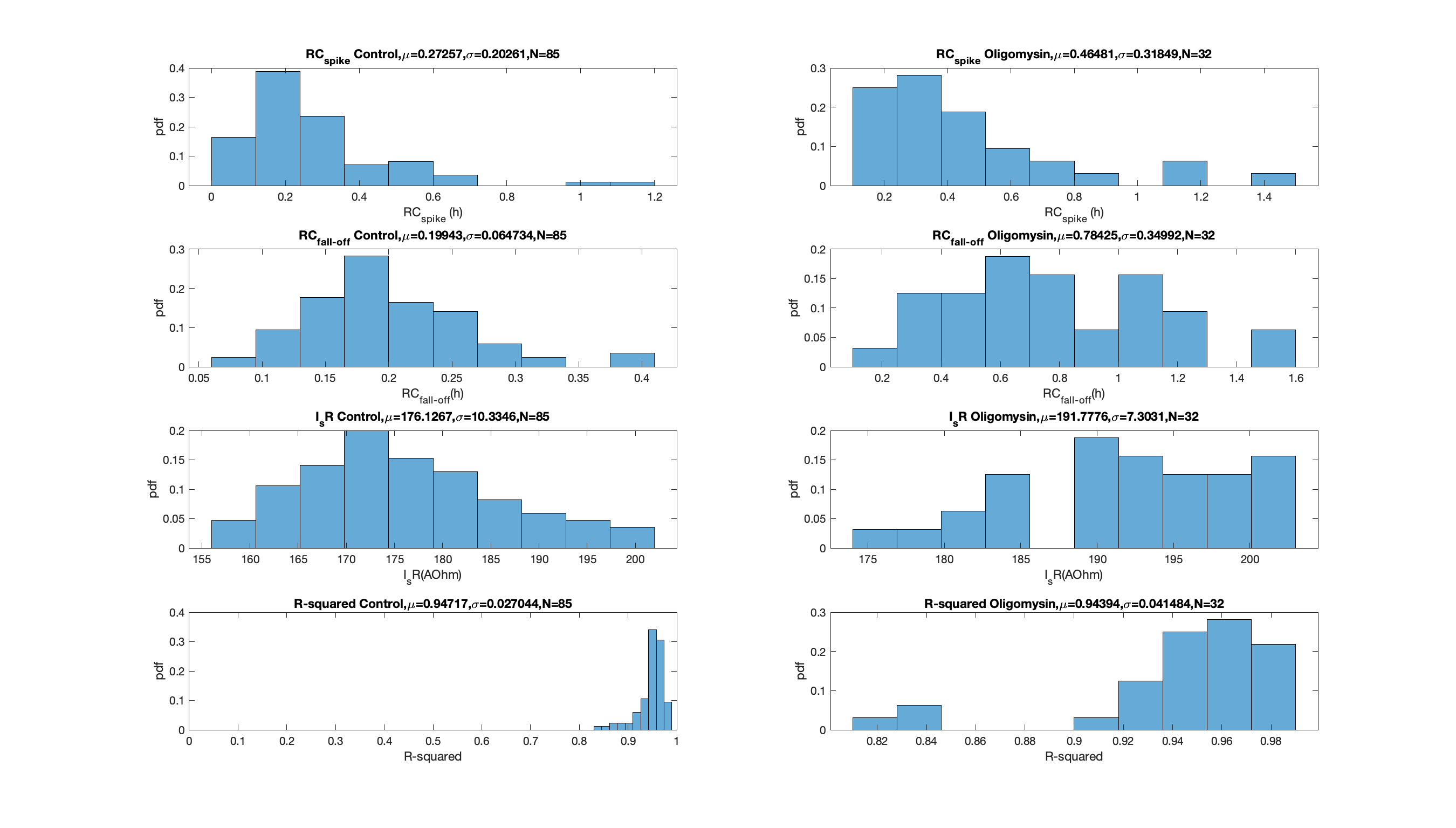


The code also provides the currents of ATP for control cells both in file:

e.g. *SubCodes/Results/I_ATP_data_beta=4.88_gamma=0.0122/Data_for_alignment_Vo150_c20.0215* as *File_1.mat* etc and images as shown in the next page. The light green data & errors display data when leakage is not assumed to change with membrane potential, whereas the dark green data displays data when leakage has a non-ohmic scaling with membrane potential.


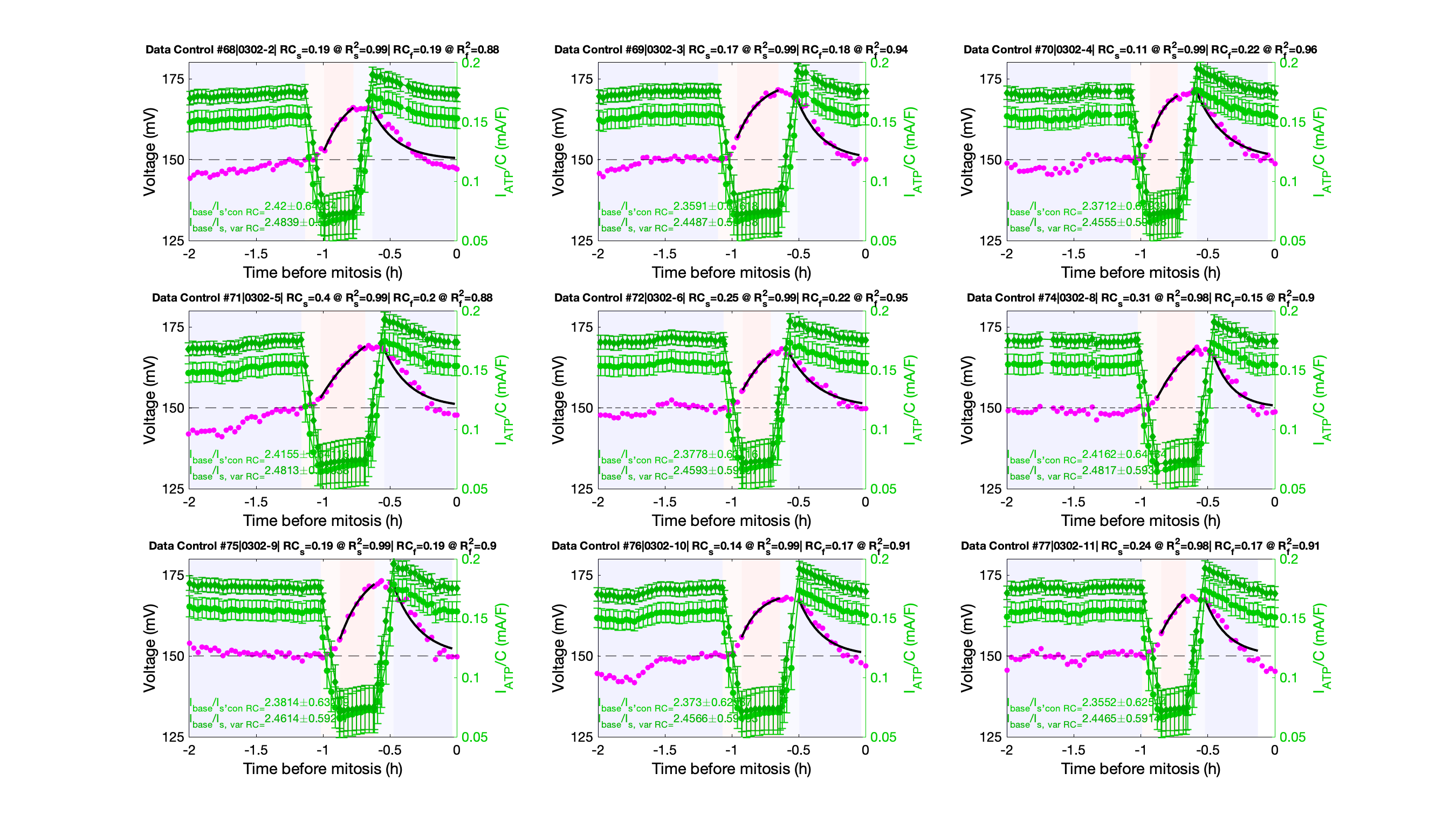


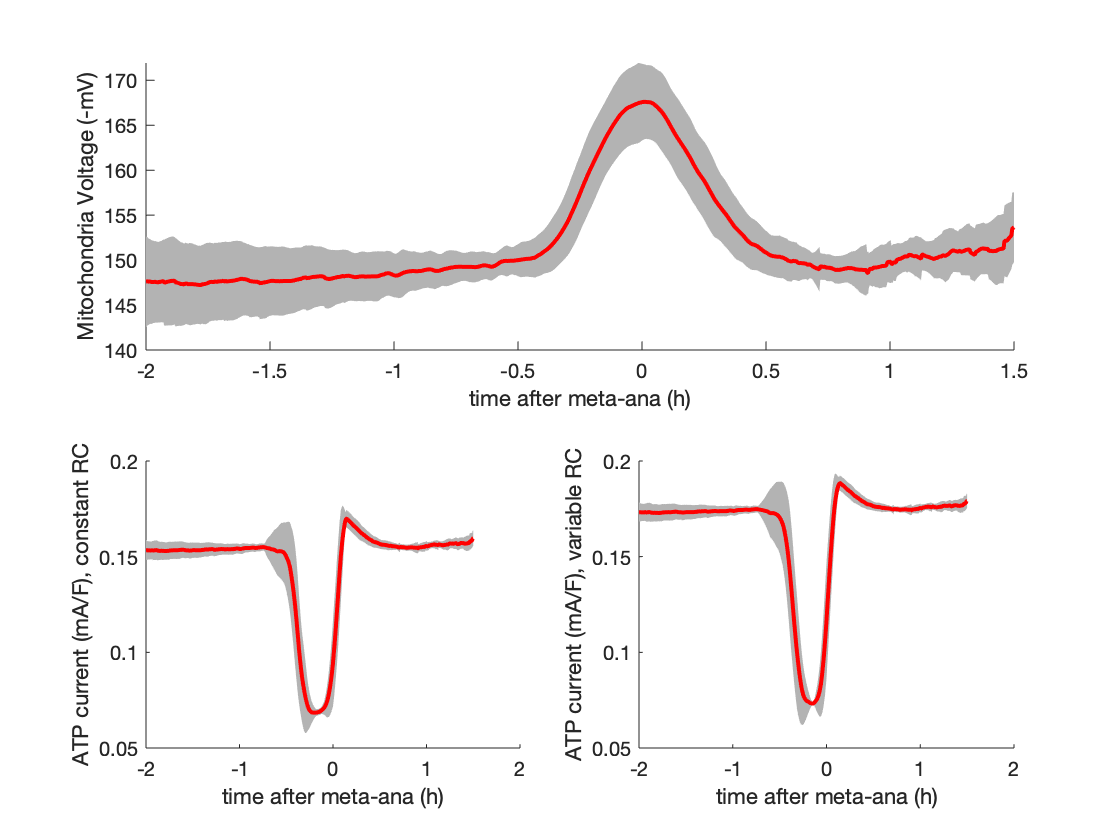
In Step 4, the code gives the figure of ATP current averaged over the control cells as shown in the following image. The shaded area represents variability between cells but does not contain error in estimation of R_ATP_.

**Additional Capabilities**

1. By default, the codes calculate fits for Δt intervals, lin_increase=8.57/60 (hours) lin_decrease=8.57/60 (hours), c2=0.0215, baseline voltage Vo=150 (mV). The user can change these variables in the lines listed in the table below. To use these values the user also needs to set the variable do_force_values equal to 1;

|  | Line # in both *Subcodes/Control/Mito_Control_analysis.m* and *Subcodes/Oligomysin/ Mito_Oligomysin_analysis.m* |
| --- | --- |
| lin_increase | 70 |
| lin_decrease | 71 |
| do_force_values | 74 |
| c2_exp_forced | 75 |
| Vo_forced | 76 |

1. By default, the codes calculate fits for a single pair (c2,Vo). The user can make multiple pairs by setting the desired range in variables shown in the table below. To use these values the user also needs to set the variable do_force_values equal to 0. The capacity of the code to do the analysis over multiple pairs of (c2,Vo) allows for sensitivity analysis over these parameters.

|  | Line # in both *Subcodes/Control/Mito_Control_analysis.m* and *Subcodes/Oligomysin/ Mito_Oligomysin_analysis.m* |
| --- | --- |
| V_Control_array | 72 |
| c2_exp_array | 73 |
| do_force_values | 74 |

1. By default, the codes use values for calculating ATP currents derived from experiments and literature: beta_vector=4.88 and gamma_vector=0.0122. The user needs to change these two variables only in *SubCodes/Control/Mito_Control_analysis.m* in the lines shown in the table below.

|  | Line # in *Subcodes/Control/Mito_Control_analysis.m* |
| --- | --- |
| beta_vector | 1244 |
| gamma_vector | 1245 |

1. By default, the codes exclude a given number of control and oligomysin datasetsThe user can change the number of datasets included or excluded by changing the index numbers in the arrays for Control and Oligomysin data, shown below:

|  | Line # in *Subcodes/Control/ Mito_Control_analysis.m* | Line # in *Subcodes/Oligomysin/ Mito_Oligomysin_analysis.m* |
| --- | --- | --- |
| array_control | 150 | n/a |
| array_Oligomysin | n/a | 148 |
