# Supplementary material for: Monitoring and modeling of lymphocytic leukemia cell bioenergetics reveals decreased ATP synthesis during cell division
Source: Nat Commun. 2020 Oct 5;11:4983. doi: 10.1038/s41467-020-18769-y (PMC7536222; doi:10.1038/s41467-020-18769-y)
Supplement: Supplementary file 4 — Reporting Summary [file 41467_2020_18769_MOESM4_ESM.pdf]

nature research

Corresponding author(s): Teemu Miettinen

Last updated by author(s): Aug 21, 2020

Reporting Summary

Nature Research wishes to improve the reproducibility of the work that we publish. This form provides structure for consistency and transparency in reporting. For further information on Nature Research policies, see Authors & Referees and the Editorial Policy Checklist.

Statistics

For all statistical analyses, confirm that the following items are present in the figure legend, table legend, main text, or Methods section.

n/a	Confirmed	
☐	☒	The exact sample size (*n*) for each experimental group/condition, given as a discrete number and unit of measurement
☐	☒	A statement on whether measurements were taken from distinct samples or whether the same sample was measured repeatedly
☐	☒	The statistical test(s) used AND whether they are one- or two-sided<br>*Only common tests should be described solely by name; describe more complex techniques in the Methods section.*
☒	☐	A description of all covariates tested
☐	☒	A description of any assumptions or corrections, such as tests of normality and adjustment for multiple comparisons
☐	☒	A full description of the statistical parameters including central tendency (e.g. means) or other basic estimates (e.g. regression coefficient) AND variation (e.g. standard deviation) or associated estimates of uncertainty (e.g. confidence intervals)
☐	☒	For null hypothesis testing, the test statistic (e.g. *F*, *t*, *r*) with confidence intervals, effect sizes, degrees of freedom and *P* value noted<br>*Give P values as exact values whenever suitable.*
☒	☐	For Bayesian analysis, information on the choice of priors and Markov chain Monte Carlo settings
☒	☐	For hierarchical and complex designs, identification of the appropriate level for tests and full reporting of outcomes
☒	☐	Estimates of effect sizes (e.g. Cohen's *d*, Pearson's *r*), indicating how they were calculated

Our web collection on statistics for biologists contains articles on many of the points above.

Software and code

Policy information about availability of computer code

Data collection	LABVIEW 2012 was used for SMR data collection. BD FACSDiva software (v8.0.1) was used for flow cytometer data collection. SoftWoRx 7.0.0 software was used for microscopy image acquisition.
Data analysis	MATLAB 2014B was used for modeling, OriginPro 8 PR0 and MATLAB 2014B were used for plotting data and for statistical tests. FlowJo V10 was used for flow cytometer data analysis. SoftWoRx 7.0.0 software was used for image deconvolution. Images were analysed using ImageJ v2.0.0-rc-69/1.52p.

For manuscripts utilizing custom algorithms or software that are central to the research but not yet described in published literature, software must be made available to editors/reviewers. We strongly encourage code deposition in a community repository (e.g. GitHub). See the Nature Research guidelines for submitting code & software for further information.

Data

Policy information about availability of data

All manuscripts must include a data availability statement. This statement should provide the following information, where applicable:

- Accession codes, unique identifiers, or web links for publicly available datasets
- A list of figures that have associated raw data
- A description of any restrictions on data availability

The source data (averages and S.D. or s.e.m.) behind all box blots and bar charts are provided as a Source Data file. Data for single-cell TMRE traces are included in the Electrical circuit analysis code file.

Field-specific reporting

Please select the one below that is the best fit for your research. If you are not sure, read the appropriate sections before making your selection.

☒ Life sciences ☐ Behavioural & social sciences ☐ Ecological, evolutionary & environmental sciences

For a reference copy of the document with all sections, see nature.com/documents/nr-reporting-summary-flat.pdf

Life sciences study design

All studies must disclose on these points even when the disclosure is negative.

Sample size	No statistical tests were used to determine the sample size. All experiment rely on three or more independent samples, which was considered sufficient to determine any major effects.
Data exclusions	Some data were excluded from the modeling, as cells did not reach a steady TMRE baseline prior to cell division. This was a pre-established exclusion criteria necessitated by our modeling approach. For full details of data exclusion, please see Supplementary note 6.
Replication	All data displayed represents at least three independent samples. The experiments used for modeling (single-cell TMRE traces) were repeated over 30 times (details found in Fig. 4). All replication experiments provided similar data.
Randomization	For single-cell SMR experiments, the cell that was measured was randomly selected by loading a population of cells in to the SMR and trapping the first large (typically G2) cell that passed through the SMR.
Blinding	No blinding was carried out, as the experiments and data analysis were carried out by same individuals, which made blinding impossible.

Reporting for specific materials, systems and methods

We require information from authors about some types of materials, experimental systems and methods used in many studies. Here, indicate whether each material, system or method listed is relevant to your study. If you are not sure if a list item applies to your research, read the appropriate section before selecting a response.

Materials & experimental systems

n/a	Involved in the study	
☐	☒	Antibodies
☐	☒	Eukaryotic cell lines
☒	☐	Palaeontology
☒	☐	Animals and other organisms
☒	☐	Human research participants
☒	☐	Clinical data

Methods

n/a	Involved in the study	
☒	☐	ChIP-seq
☐	☒	Flow cytometry
☒	☐	MRI-based neuroimaging

Antibodies

Antibodies used	p-Histone H3 (Ser10) (monoclonal rabbit antibody, D2C8, conjugated to Alexa 488, Cell Signaling Technology, #3465S, batch#13)<br>MPM2 (monoclonal mouse antibody, EMD Millipore, #05-368, batch#3250291)<br>β-tubulin III (polyclonal rabbit antibody, Sigma-Aldrich, #T2200, batch#0000084267)<br>Human CD28 (monoclonal mouse antibody, BioLegend, #302901)<br>Anti-rabbit secondary antibody IRDye 680RD (goat, LI-COR, #925-68071)<br>Anti-mouse secondary antibody IRDye 800CW (donkey, LI-COR, #925-32212)<br>Anti-mouse secondary antibody conjugated to Alexa Fluor 488 (F(ab')2 Fragment antibody, Cell Signaling Technology, #4408)
Validation	p-Histone H3 (Ser10) antibody: Microscopy analysis to validate that the antibody labeling is localized on DNA in mouse L1210 cells; FACS analysis with DNA co-staining to validate cell cycle timing of the antibody target phosphorylation in mouse L1210 cells; FACS analysis following RO-3306 mediated CDK1 inhibition to validate that blockade of mitosis removes the antibody labeling in mouse L1210 cells.<br><br>MPM2 antibody: FACS and western blot analysis following RO-3306 mediated CDK1 inhibition to validate that blockade of CDK1 removes the antibody labeling in mouse L1210 cells; western blotting to validate that the antibody labeling is not dominated by single target protein in mouse L1210 cells.<br><br>β-tubulin III antibody: western blot analysis to verify correct target protein size in mouse L1210 cells.<br><br>Human CD28 antibody: No validations.

Eukaryotic cell lines

Policy information about cell lines

Cell line source(s)	L1210 cells were obtained from ATCC (Cat# CCL-219), BaF3 cells were obtained from RIKEN BioResource Center (Cat# RCB4476), DT40 cells were a gracious gift from K. Samejima and B. Earnshaw from University of Edinburgh (original source unknown), Fl5.12 cells were a gracious gift from M. Vander Heiden from Massachusetts Institute of Technology (original source unknown), S-HeLa cells were a gracious gift from K. Elias from Brigham Women's Hospital (original source unknown), and HEK293T cells were obtained from ATCC (Cat# CRL-3216).
Authentication	L1210 and BaF3 cell lines were authenticated by supplier company. In addition, L1210 cells were authenticated by visual inspection and by comparing transcriptional profiles to previously published profiles. S-Hela, Fl5.12 and DT40 cells were authenticated only by visual inspection.
Mycoplasma contamination	All cell lines were tested to be free of mycoplasma.
Commonly misidentified lines (See ICLAC register)	No commonly misidentified cell lines were used.

Flow Cytometry

Plots

Confirm that:

☒ The axis labels state the marker and fluorochrome used (e.g. CD4-FITC).

☒ The axis scales are clearly visible. Include numbers along axes only for bottom left plot of group (a 'group' is an analysis of identical markers).

☒ All plots are contour plots with outliers or pseudocolor plots.

☒ A numerical value for number of cells or percentage (with statistics) is provided.

Methodology

Sample preparation	For cell cycle analysis, the cells were washed with PBS, fixed in 4 % PFA for 10 min, washed with PBS, permeabilized with 0.5 % Triton X-100 for 10 min, washed with PBS and blocked with 5 % BSA in PBS for 30 min. The cells were then stained with p-Histone H3 (S10) antibody (D2C8, conjugated to Alexa 488, Cell Signaling Technology, #3465S) in a PBS solution containing 5 % BSA o/n at +4°C. The p-Histone H3 antibody was used in the concentration recommended by the supplier. The following day the cells were washed with PBS and stained with 1:2000 dilution of NuclearMask Blue (#H10325, Thermo Fisher Scientific) for 30 min in RT. Finally, the cells were washed three times with PBS, mixed in to PBS supplemented with 1% BSA and put on ice until FACS analysis.
Instrument	BD Biosciences flow cytometer LSR II HTS with excitation lasers at 355 nm, 488 nm and 561 nm, and emission filters at 450/50, 530/30 and 585/15.
Software	FlowJo software was used for data analysis.
Cell population abundance	For cell cycle experiments, each analysed subpopulation contained at least 1,000 cells. For sorting of transfected cells, at least 10,000 cells with a high expression of the transfected construct (fluorescent metabolic reporter, such as A-Team ATP sensor) were sorted out and the purity of the sorting was validated later by counting the relative abundance of fluorescent cells.
Gating strategy	FSC and SSC gating was used to exclude particles smaller than any viable cell and to carry out doublet discrimination. Gating of mitotic cells, G2 cells and G1 cells was done based on p-Histone S10 and DNA labeling, as shown in Figures S9A,B.

☒ Tick this box to confirm that a figure exemplifying the gating strategy is provided in the Supplementary Information.
